# Physical Activity Attenuates the Influence of *FTO* Variants on Obesity Risk: A Meta-Analysis of 218,166 Adults and 19,268 Children

**DOI:** 10.1371/journal.pmed.1001116

**Published:** 2011-11-01

**Authors:** Tuomas O. Kilpeläinen, Lu Qi, Soren Brage, Stephen J. Sharp, Emily Sonestedt, Ellen Demerath, Tariq Ahmad, Samia Mora, Marika Kaakinen, Camilla Helene Sandholt, Christina Holzapfel, Christine S. Autenrieth, Elina Hyppönen, Stéphane Cauchi, Meian He, Zoltan Kutalik, Meena Kumari, Alena Stančáková, Karina Meidtner, Beverley Balkau, Jonathan T. Tan, Massimo Mangino, Nicholas J. Timpson, Yiqing Song, M. Carola Zillikens, Kathleen A. Jablonski, Melissa E. Garcia, Stefan Johansson, Jennifer L. Bragg-Gresham, Ying Wu, Jana V. van Vliet-Ostaptchouk, N. Charlotte Onland-Moret, Esther Zimmermann, Natalia V. Rivera, Toshiko Tanaka, Heather M. Stringham, Günther Silbernagel, Stavroula Kanoni, Mary F. Feitosa, Soren Snitker, Jonatan R. Ruiz, Jeffery Metter, Maria Teresa Martinez Larrad, Mustafa Atalay, Maarit Hakanen, Najaf Amin, Christine Cavalcanti-Proença, Anders Grøntved, Göran Hallmans, John-Olov Jansson, Johanna Kuusisto, Mika Kähönen, Pamela L. Lutsey, John J. Nolan, Luigi Palla, Oluf Pedersen, Louis Pérusse, Frida Renström, Robert A. Scott, Dmitry Shungin, Ulla Sovio, Tuija H. Tammelin, Tapani Rönnemaa, Timo A. Lakka, Matti Uusitupa, Manuel Serrano Rios, Luigi Ferrucci, Claude Bouchard, Aline Meirhaeghe, Mao Fu, Mark Walker, Ingrid B. Borecki, George V. Dedoussis, Andreas Fritsche, Claes Ohlsson, Michael Boehnke, Stefania Bandinelli, Cornelia M. van Duijn, Shah Ebrahim, Debbie A. Lawlor, Vilmundur Gudnason, Tamara B. Harris, Thorkild I. A. Sørensen, Karen L. Mohlke, Albert Hofman, André G. Uitterlinden, Jaakko Tuomilehto, Terho Lehtimäki, Olli Raitakari, Bo Isomaa, Pål R. Njølstad, Jose C. Florez, Simin Liu, Andy Ness, Timothy D. Spector, E. Shyong Tai, Philippe Froguel, Heiner Boeing, Markku Laakso, Michael Marmot, Sven Bergmann, Chris Power, Kay-Tee Khaw, Daniel Chasman, Paul Ridker, Torben Hansen, Keri L. Monda, Thomas Illig, Marjo-Riitta Järvelin, Nicholas J. Wareham, Frank B. Hu, Leif C. Groop, Marju Orho-Melander, Ulf Ekelund, Paul W. Franks, Ruth J. F. Loos

**Affiliations:** 1 Medical Research Council Epidemiology Unit, Institute of Metabolic Science, Cambridge, United Kingdom; 2 Departments of Epidemiology and Nutrition, Harvard University School of Public Health, Boston, Massachusetts, United States of America; 3 Lund University Diabetes Centre, Department of Clinical Sciences, Skane University Hospital, Lund University, Malmo, Sweden; 4 Division of Epidemiology and Community Health, University of Minnesota, Minneapolis, Minnesota, United States of America; 5 Division of Cardiology, Department of Medicine, Duke University Medical Center, Durham, North Carolina, United States of America; 6 Brigham and Women’s Hospital, Harvard Medical School, Boston, Massachusetts, United States of America; 7 Institute of Health Sciences and Biocenter Oulu, University of Oulu, Oulu, Finland; 8 Hagedorn Research Institute, Gentofte, Denmark; 9 Unit for Molecular Epidemiology, Helmholtz Zentrum München—German Research Center for Environmental Health, Neuherberg, Germany; 10 Else Kroener-Fresenius-Centre for Nutritional Medicine, Technische Universität München, University Hospital “Klinikum rechts der Isar,” Munich, Germany; 11 Institute of Epidemiology II, Helmholtz Zentrum München—German Research Center for Environmental Health, Neuherberg, Germany; 12 Centre for Paediatric Epidemiology and Biostatistics and Medical Research Council Centre of Epidemiology for Child Health, University College London Institute of Child Health, London, United Kingdom; 13 CNRS-UMR-8090, Department of Genomics and Molecular Physiology of Metabolic Diseases, Institute of Biology of Lille, Lille, France; 14 Harvard School of Public Health, Boston, Massachusetts, United States of America; 15 Department of Medical Genetics, University of Lausanne, Lausanne, Switzerland; 16 Genetic Epidemiology Group, Department of Epidemiology, University College London, London, United Kingdom; 17 Department of Medicine, University of Eastern Finland and Kuopio University Hospital, Kuopio, Finland; 18 Department of Epidemiology, German Institute of Human Nutrition Potsdam-Rehbrücke, Nuthetal, Germany; 19 INSERM, CESP Centre for Research in Epidemiology and Population Health, U1018, Epidemiology of diabetes, obesity and chronic kidney disease over the lifecourse and determinants of early nutrition, Villejuif, France; 20 University Paris Sud 11, UMRS 1018, Villejuif, France; 21 Department of Epidemiology and Public Health, Yong Loo Lin School of Medicine, National University of Singapore, Singapore; 22 King’s College London, London, United Kingdom; 23 Medical Research Council Centre for Causal Analyses in Translational Epidemiology, School of Social and Community Medicine, University of Bristol, Bristol, United Kingdom; 24 Division of Preventive Medicine, Brigham and Women’s Hospital, Harvard Medical School, Boston, Massachusetts, United States of America; 25 Department of Internal Medicine, Erasmus MC, Rotterdam, The Netherlands; 26 Netherlands Genomics Initiative–sponsored Netherlands Consortium for Healthy Aging, Leiden, The Netherlands; 27 The Biostatistics Center, George Washington University, Rockville, Maryland, United States of America; 28 National Institute on Aging, National Institutes of Health, Bethesda, Maryland, United States of America; 29 Center for Medical Genetics and Molecular Medicine, Haukeland University Hospital, Bergen, Norway; 30 Department of Clinical Medicine, University of Bergen, Bergen, Norway; 31 Department of Biostatistics and Center for Statistical Genetics, University of Michigan, Ann Arbor, Michigan, United States of America; 32 Department of Genetics, University of North Carolina, Chapel Hill, North Carolina, United States of America; 33 Molecular Genetics Section, Department of Pathology and Medical Biology, University Medical Centre Groningen and University of Groningen, Groningen, The Netherlands; 34 Complex Genetics Section, Department of Medical Genetics, University Medical Center Utrecht, Utrecht, The Netherlands; 35 Julius Center for Health Sciences and Primary Care, University Medical Center Utrecht, Utrecht, The Netherlands; 36 Institute of Preventive Medicine, Copenhagen University Hospital, Copenhagen, Denmark; 37 Institute of Biomedical Science, Faculty of Health Sciences, University of Copenhagen, Copenhagen, Denmark; 38 Genetic Epidemiology Unit, Department of Epidemiology, Erasmus MC, Rotterdam, The Netherlands; 39 Medstar Research Institute, Baltimore, Maryland, United States of America; 40 Longitudinal Study Section, National Institute on Aging, National Institutes of Health, Baltimore, Maryland, United States of America; 41 Department of Internal Medicine, Division of Endocrinology, Diabetology, Nephrology, Vascular Disease, and Clinical Chemistry, Eberhard-Karls-University Tübingen, Tübingen, Germany; 42 Department of Nutrition-Dietetics, Harokopio University of Athens, Athens, Greece; 43 Division of Statistical Genomics, Washington University School of Medicine, St. Louis, Missouri, United States of America; 44 University of Maryland School of Medicine, College Park, Maryland, United States of America; 45 Unit for Preventive Nutrition, Department of Biosciences and Nutrition at NOVUM, Karolinska Institutet, Huddinge, Sweden; 46 Department of Physical Education and Sport, School of Physical Activity and Sport Sciences, University of Granada, Granada, Spain; 47 Centro de Investigación Biomédica en Red de Diabetes y Enfermedades Metabólicas Asociadas, Hospital Clínico San Carlos, Madrid, Spain; 48 Institute of Biomedicine, Department of Physiology, University of Eastern Finland, Kuopio Campus, Kuopio, Finland; 49 The Research Centre of Applied and Preventive Cardiovascular Medicine, University of Turku, Turku, Finland; 50 Institute of Sport Science and Clinical Biomechanics, University of Southern Denmark, Odense, Denmark; 51 Department of Public Health and Clinical Medicine Section for Nutritional Research, Umeå University, Umeå, Sweden; 52 Department of Physiology, Institute of Neuroscience and Physiology, Sahlgrenska Academy, University of Gothenburg, Gothenburg, Sweden; 53 Department of Clinical Physiology, University of Tampere and Tampere University Hospital, Tampere, Finland; 54 Steno Diabetes Centre, Gentofte, Denmark; 55 Faculty of Health Sciences, University of Aarhus, Aarhus, Denmark; 56 Marie Krogh Center for Metabolic Research, Faculty of Health Sciences, University of Copenhagen, Copenhagen, Denmark; 57 Division of Kinesiology, Department of Social and Preventive Medicine, Laval University, Ste-Foy, Quebec, Canada; 58 Genetic Epidemiology and Clinical Research Group, Department of Public Health and Clinical Medicine, Section for Medicine, Umeå University Hospital, Umeå, Sweden; 59 Department of Odontology, Umeå University, Umeå, Sweden; 60 Department of Medical Statistics, London School of Hygiene and Tropical Medicine, London, United Kingdom; 61 Finnish Institute of Occupational Health, Oulu, Finland; 62 LIKES Research Center for Sport and Health Sciences, Jyväskylä, Finland; 63 Department of Medicine, University of Turku, Turku, Finland; 64 Institute of Public Health and Clinical Nutrition, University of Eastern Finland, Kuopio Campus, Kuopio, Finland; 65 Research Unit, Kuopio University Hospital, Kuopio, Finland; 66 Human Genomics Laboratory, Pennington Biomedical Research Center, Baton Rouge, Louisiana, United States of America; 67 INSERM, U744, Institut Pasteur de Lille, Université Lille Nord de France, Université Lille 2, Lille, France; 68 Division of Endocrinology, Diabetes and Nutrition, University of Maryland School of Medicine, Baltimore, Maryland, United States of America; 69 Institute of Cell and Molecular Biosciences, Newcastle University, Newcastle, United Kingdom; 70 Centre for Bone and Arthritis Research, Department of Internal Medicine, Institute of Medicine, Sahlgrenska Academy, University of Gothenburg, Gothenburg, Sweden; 71 Geriatric Rehabilitation Unit, Azienda Sanitaria Firenze, Florence, Italy; 72 Netherlands Genomics Initiative, Centre for Medical Systems Biology, Leiden, The Netherlands; 73 Faculty of Epidemiology and Population Health, London School of Hygiene and Tropical Medicine, London, United Kingdom; 74 Icelandic Heart Association, Heart Preventive Clinic and Research Institute, Kopavogur, Iceland; 75 University of Iceland, Reykjavik, Iceland; 76 Intramural Research Program, National Institute on Aging, National Institutes of Health, Bethesda, Maryland, United States of America; 77 Hjelt Institute, Department of Public Health, University of Helsinki, Helsinki, Finland; 78 South Ostrobothnia Central Hospital, Seinäjoki, Finland; 79 Department of Clinical and Preventive Medicine, Danube-University Krems, Krems, Austria; 80 Department of Clinical Chemistry, University of Tampere and Tampere University Hospital, Tampere, Finland; 81 Department of Clinical Physiology, Turku University Hospital, Turku, Finland; 82 Folkhälsan Research Centre, Helsinki, Finland; 83 Department of Social Services and Health Care, Jakobstad, Finland; Department of Pediatrics, Haukeland University Hospital, Bergen, Norway; 85 Center for Human Genetic Research and Diabetes Research Center, Massachusetts General Hospital, Boston, Massachusetts, United States of America; 86 Department of Medicine, Harvard Medical School, Boston, Massachusetts, United States of America; 87 Program for Medical and Population Genetics, Broad Institute, Cambridge, Massachusetts, United States of America; 88 Center for Metabolic Disease Prevention, School of Public Health and David Geffen School of Medicine, University of California, Los Angeles, California, United States of America; 89 School of Oral and Dental Sciences, University of Bristol, Bristol, United Kingdom; 90 Department of Medicine, Yong Loo Lin School of Medicine, National University of Singapore, Singapore; 91 Centre and Department of Genomic Medicine, Hammersmith Hospital, Imperial College London, London, United Kingdom; 92 Department of Epidemiology and Public Health, University College London, London, United Kingdom; 93 Department of Public Health and Primary Care, Institute of Public Health, University of Cambridge, Cambridge, United Kingdom; 94 Faculty of Health Sciences, University of Southern Denmark, Odense, Denmark; 95 Department of Epidemiology, University of North Carolina, Chapel Hill, North Carolina, United States of America; 96 Department of Epidemiology and Biostatistics, Imperial College London, London, United Kingdom; 97 Department of Life Course and Services, National Institute for Health and Welfare, Oulu, Finland; Kings College London, United Kingdom

## Abstract

**Background:**

The *FTO* gene harbors the strongest known susceptibility locus for obesity. While many individual studies have suggested that physical activity (PA) may attenuate the effect of *FTO* on obesity risk, other studies have not been able to confirm this interaction. To confirm or refute unambiguously whether PA attenuates the association of *FTO* with obesity risk, we meta-analyzed data from 45 studies of adults (*n = *218,166) and nine studies of children and adolescents (*n = *19,268).

**Methods and Findings:**

All studies identified to have data on the *FTO* rs9939609 variant (or any proxy [*r* ^2^>0.8]) and PA were invited to participate, regardless of ethnicity or age of the participants. PA was standardized by categorizing it into a dichotomous variable (physically inactive versus active) in each study. Overall, 25% of adults and 13% of children were categorized as inactive. Interaction analyses were performed within each study by including the *FTO*×PA interaction term in an additive model, adjusting for age and sex. Subsequently, random effects meta-analysis was used to pool the interaction terms. In adults, the minor (A−) allele of rs9939609 increased the odds of obesity by 1.23-fold/allele (95% CI 1.20–1.26), but PA attenuated this effect (*p*_interaction_  = 0.001). More specifically, the minor allele of rs9939609 increased the odds of obesity less in the physically active group (odds ratio  = 1.22/allele, 95% CI 1.19–1.25) than in the inactive group (odds ratio  = 1.30/allele, 95% CI 1.24–1.36). No such interaction was found in children and adolescents.

**Conclusions:**

The association of the *FTO* risk allele with the odds of obesity is attenuated by 27% in physically active adults, highlighting the importance of PA in particular in those genetically predisposed to obesity.

*Please see later in the article for the Editors' Summary*

## Abstract 2

Ruth Loos and colleagues report findings from a meta-analysis of multiple studies examining the extent to which physical activity attenuates effects of a specific gene variant, FTO, on obesity in adults and children. They report a fairly substantial attenuation by physical activity on the effects of this genetic variant on the risk of obesity in adults.

## Editors’ Summary

**Background:**

Two in three Americans are overweight, of whom half are obese, and the trend towards increasing obesity is now seen across developed and developing countries. There has long been interest in understanding the impact of genes and environment when it comes to apportioning responsibility for obesity. Carrying a change in the *FTO* gene is common (found in three-quarters of Europeans and North Americans) and is associated with a 20%–30% increased risk of obesity. Some overweight or obese individuals may feel that the dice are loaded and there is little point in fighting the fat; it has been reported that those made aware of their genetic susceptibility to obesity may still choose a poor diet. A similar fatalism may occur when overweight and obese people consider physical activity. But disentangling the influence of physical activity on those genetically susceptible to obesity from other factors that might impact weight is not straightforward, as it requires large sample sizes, could be subject to publication bias, and may rely on less than ideal self-reporting methods.

**Why Was This Study Done?:**

The public health ramifications of understanding the interaction between genetic susceptibility to obesity and physical activity are considerable. Tackling the rising prevalence of obesity will inevitably include interventions principally aimed at changing dietary intake and/or increasing physical activity, but the evidence for these with regards to those genetically susceptible has been lacking to date. The authors of this paper set out to explore the interaction between the commonest genetic susceptibility trait and physical activity using a rigorous meta-analysis of a large number of studies.

**What Did the Researchers Do and Find?:**

The authors were concerned that a meta-analysis of published studies would be limited both by the data available to them and by possible bias. Instead of this more widely used approach, they took the literature search as their starting point, identified other studies through their collaborators’ network, and then undertook a meta-analysis of all available studies using a new and standardized analysis plan. This entailed an extremely large number of authors mining their data afresh to extract the relevant data points to enable such a meta-analysis. Physical activity was identified in the original studies in many different ways, including by self-report or by using an external measure of activity or heart rate. In order to perform the meta-analysis, participants were labeled as physically active or inactive in each study. For studies that had used a continuous scale, the authors decided that the bottom 20% of the participants were inactive (10% for children and adolescents). Using data from over 218,000 adults, the authors found that carrying a copy of the susceptibility gene increased the odds of obesity by 1.23-fold. But the size of this influence was 27% less in the genetically susceptible adults who were physically active (1.22-fold) compared to those who were physically inactive (1.30-fold). In a smaller study of about 19,000 children, no such effect of physical activity was seen.

**What Do these Findings Mean?:**

This study demonstrates that people who carry the susceptibility gene for obesity can benefit from physical activity. This should inform health care professionals and the wider public that the view of genetically determined obesity not being amenable to exercise is incorrect and should be challenged. Dissemination, implementation, and ensuring uptake of effective physical activity programs remains a challenge and deserves further consideration. That the researchers treated “physically active” as a yes/no category, and how they categorized individuals, could be criticized, but this was done for pragmatic reasons, as a variety of means of assessing physical activity were used across the studies. It is unlikely that the findings would have changed if the authors had used a different method of defining physically active. Most of the studies included in the meta-analysis looked at one time point only; information about the influence of physical activity on weight changes over time in genetically susceptible individuals is only beginning to emerge.

**Additional Information:**

Please access these websites via the online version of this summary at http://dx.doi.org/10.1371/journal.pmed.1001116.

- This study is further discussed in a *PLoS Medicine* Perspective by Lennert Veerman
- The US Centers for Disease Control and Prevention provides obesity-related statistics, details of prevention programs, and an overview on public health strategy in the United States
- A more worldwide view is given by the World Health Organization
- The UK National Health Service website gives information on physical activity guidelines for different age groups, while similar information can also be found from US sources

## Introduction

Over the past three decades, there has been a global increase in the prevalence of obesity, which has been mainly driven by changes in lifestyle [1]. However, not everyone becomes obese in today’s obesogenic environment. In fact, twin studies suggest that changes in adiposity in response to environmental influences are genetically determined [2]–[6]. Until recently, there were no confirmed obesity-susceptibility loci that could be used to test whether the influence of genetic susceptibility on obesity risk is enhanced by an unhealthy lifestyle. However, in 2007, the intron 1 of the fat mass and obesity associated (*FTO*) gene was identified as the first robust obesity-susceptibility locus in genome-wide association studies [7],[8]. Each additional minor allele of the rs9939609 single nucleotide polymorphism (SNP) in *FTO* was found to be associated with a 20%–30% increase in the risk of obesity and a 1–1.5 kg increase in body weight [7],[8]. The risk-increasing allele of *FTO* is common, with 74% of individuals of European descent (HapMap CEU population), 76% of individuals of African-American descent (HapMap ASW population), and 28%–44% of individuals of Asian descent (HapMap CHB, CHD, GIH, and JPT populations) carrying at least one copy of the *FTO* risk allele.

After the discovery of *FTO*, several studies reported that its obesity-increasing effect may be attenuated in individuals who are physically active [9]–[20]. Other studies, however, were unable to replicate this interaction [21]–[26], leaving it unresolved whether physical activity (PA) can reduce *FTO*’s effect on obesity risk, and if so, to what extent. Identifying interactions between genetic variants and lifestyle is challenging as it requires much larger sample sizes than those needed for the detection of main effects of genes or environment [27]. Interaction studies are further hampered by the difficulty of measuring lifestyle exposures accurately, which reduces statistical power and necessitates large study sample sizes to offset this effect [28].

To collect a sufficiently large sample to unambiguously confirm or refute the interaction between *FTO* and PA, we meta-analyzed data from 45 studies, totaling 218,166 adults. In addition, we performed a similar meta-analysis among 19,268 children and adolescents from nine studies. We included all available data, both published and unpublished, and used standardized methods to analyze the interaction across the studies.

## Methods

### Ethics Statement

All studies were conducted according to the Declaration of Helsinki. Informed consent was obtained from all participants, and the studies were approved by the ethics committees of the participating institutions.

### Study Design

The literature on the interaction between *FTO* and PA is inconsistent with regard to the definitions used for PA, the statistical analysis of interactions, and the presentation of interaction results [29]. Furthermore, as statistically non-significant interactions may often not be reported, a meta-analysis of only published results would suffer from publication bias [29]. Therefore, a literature-based meta-analysis of the interaction between *FTO* and PA was not considered appropriate. Instead, we designed a meta-analysis based on de novo analyses of data according to a standardized plan to achieve the greatest consistency possible across studies, and to allow inclusion of all available studies, irrespective of whether they had or had not been used to examine this hypothesis in the past.

We identified all eligible studies by a PubMed literature search in December 2009 using the search term “FTO” and by following the publication history of each identified study to find those with data on PA. Furthermore, we identified all studies with genome-wide association data from published papers of genome-wide association consortia by a PubMed search using the keywords “genome-wide” and “association,” and searched the literature to determine whether these studies also had data on PA. Additional yet-unpublished studies were identified through the network of collaborators who joined the meta-analysis and were also included in the meta-analysis. Analyses according to our standardized plan were performed by each study locally, and detailed summary statistics were subsequently submitted using our standardized data collection form. Alternatively, datasets were sent to us to perform the required analyses centrally (15 studies) (Tables S1 and S2).

### Quality Control

The data collection form included questions that allowed testing for internal consistency. The data were extracted automatically and cross-checked manually. The same checks for internal consistency were performed independent of whether the data were analyzed locally or centrally. All ambiguities were clarified with the respective study investigators before the final meta-analyses. A funnel plot, along with Begg and Egger tests, was used to test for the presence of “positive results bias” (i.e., to test whether studies with positive results were more likely to participate in the meta-analysis than those with negative or inconclusive results) (Figure S1).

### Standardization of Physical Activity

PA was measured in various ways across the participating studies of the meta-analysis. Therefore, we standardized PA by categorizing it into a dichotomous variable (physically inactive versus active) in each study. In studies with categorical PA data, adults were defined as being “inactive” when they had a sedentary occupation and if they reported less than 1 h of moderate-to-vigorous leisure-time or commuting PA per week. In studies with continuous data on PA, adults were defined as being “inactive” when their level of PA was in the lowest sex-specific 20% of the study population concerned. All other individuals were defined as “physically active.” For children and adolescents, a more stringent cut-off for “inactivity” was chosen than for adults because of the high average PA levels in younger children [30] and the known weaker association between PA and childhood body mass index (BMI) [31]. Thus, children and adolescents were defined as being “inactive” when their level of PA was in the lowest sex- and age-specific 10% of the study population. The coding of the dichotomous PA variable in each study is described in detail in Text S1.

### Genotyping

The rs9939609 SNP or a proxy (linkage disequilibrium *r* ^2^>0.8 in the corresponding ethnic group) was genotyped in each study using either direct genotyping methods or Affymetrix and Illumina genome-wide genotyping arrays (Text S1). The studies submitted only data that met their quality control criteria for genotyping call rate, concordance in duplicate samples, and Hardy-Weinberg Equilibrium *p-*value (Text S1).

### Measurement of BMI, Waist Circumference, and Body Fat Percentage

BMI was calculated in each study by dividing height (in meters) by weight (in kilograms) squared. Waist circumference was measured with standard protocols and was not adjusted for height in the analyses. Body fat percentage was measured using dual energy X-ray absorptiometry (seven studies), bioimpedance (11 studies), or the sum of skinfolds (seven studies) (Tables S3 and S4).

### *FTO*×PA Interaction Analysis in Participating Studies

Each study tested for an effect of the *FTO*×PA interaction on BMI, waist circumference, and body fat percentage using the following additive genetic model: 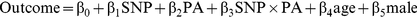
(1)


The same model was used to test for an effect of the *FTO*×PA interaction on the odds of obesity (BMI≥30 versus BMI<25 kg/m^2^) and overweight (BMI≥25 versus BMI<25 kg/m^2^) in adults, using normal-weight individuals as the reference group and testing for an additive effect in the “log odds” scale. In addition, each study tested the main effect of the *FTO* SNP on each outcome in the whole study population and in the inactive and physically active subgroups separately, using the model 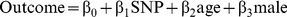
(2)


Each study also tested the main effect of PA on each outcome, using the model 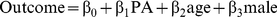
(3)


The interactions and associations of continuous outcome variables were analyzed with linear regression and those of dichotomous variables with logistic regression. In adults, BMI, waist circumference, and body fat percentage were analyzed as non-transformed variables, whereas in children, age- and sex-specific *Z*-scores of BMI, waist circumference, and body fat percentage were used.

Where data were from case-control studies for any outcome (Tables S1 and S2), cases and controls were analyzed separately. In studies with multiple ethnicities, each ethnicity was analyzed separately.

### Meta-Analysis and Meta-Regression

Because of heterogeneity between the studies participating in the meta-analysis, we pooled beta coefficients and standard errors for the main and interaction effects from individual studies using “DerSimonian and Laird” random effects meta-analysis, implemented by the *metan* command in Stata, version 11 (StataCorp). To confirm that our results were robust, we additionally pooled the interaction effects using the “Mantel and Haenszel” fixed effects method in Stata. However, as beta coefficients of fixed effects models and random effects models were the same (to two decimal points’ accuracy) for all traits, we report only the results for the random effects models. Data from adults and children were meta-analyzed separately. In all meta-analyses, between-study heterogeneity was tested by the *Q* statistic and quantified by the *I* ^2^ value. Low heterogeneity was defined as an *I* ^2^ value of 0%–25%, moderate heterogeneity as an *I* ^2^ of 25%–75%, and high heterogeneity as an *I* ^2^ of 75%–100% [32].

We performed a meta-regression to explore sources of heterogeneity in our meta-analysis using the *metareg* command in Stata. Meta-regression included the following study-specific variables as covariates: study sample size, proportion of inactive individuals, age (mean age or age group <60 y versus ≥60 y), sex (male versus female), mean BMI, study design (population- or family-based versus case-control), self-reported ethnicity (white, African American, Asian, Hispanic), geographic region (North America, Europe, Asia), and measurement of PA (1: studies with a continuous PA variable versus studies with categorical data; 2: measurement of both occupational and leisure-time PA versus leisure-time PA only; 3: measurement of PA with a questionnaire versus objective measurement).

Differences in interaction effect sizes between two subgroups were assessed with a *t*-test.

## Results

### Studies Included

**Figure 1:**
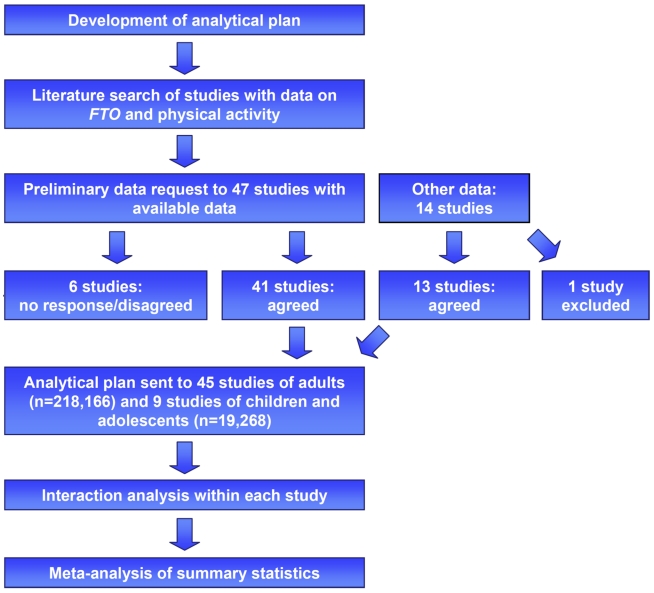
Study design of the *FTO*×PA interaction meta-analysis. Eligible studies were identified by a literature search, as well as through personal contacts (indicated in the figure as “other data”). Of all studies that were invited, 45 studies of adults (*n = *218,166) and nine studies of children and adolescents (*n = *19,268) joined the meta-analysis. A standardized analytical plan was sent to each of the studies. Summary statistics were subsequently meta-analyzed.

Our literature search identified 47 studies with data available on *FTO* and PA, of which 41 agreed to participate in our meta-analysis (Figure 1). Furthermore, 14 additional yet-unpublished studies that were identified through the network of collaborators who joined the meta-analysis were also invited to participate in the meta-analysis. We excluded one of these studies, however, because of inadequate measurement of PA. Eventually, our final meta-analysis comprised cross-sectional data on 218,166 adults (209,118 whites, 6,309 Asians, 1,770 African-Americans, and 969 Hispanics) from 45 studies, as well as data on 19,268 white children and adolescents from nine studies. Of the 45 studies of adults, 33 were from Europe, ten from North America, and two from Asia. All studies of children and adolescents were from Europe. In each study, PA was assessed using a self-report questionnaire, an accelerometer, or a heart rate sensor (Text S1).

### Association of Physical Activity with Obesity Traits (Main Effects)

Physically active adults had a 33% lower odds of obesity (*p = *2×10^−13^), 19% lower odds of overweight (*p = *7×10^−9^), 0.79 kg/m^2^ lower BMI (*p = *3×10^−15^), 2.44 cm smaller waist circumference (*p = *1×10^−20^), and 1.30% lower body fat percentage (*p = *2×10^−15^) than inactive adults (Table S1). In children, PA did not have a statistically significant association with age- and sex-standardized BMI (*p = *0.2), but physically active children had a waist circumference −0.11 *Z*-score units smaller (*p = *0.04) and a body fat percentage −0.21 *Z*-score units lower (*p = *0.02) than inactive children (Table S2).

### Association of *FTO* with Obesity Traits (Main Effects)

In adults, each additional risk allele of the *FTO* rs9939609 variant increased the odds of obesity and overweight by 23% (*p = *7×10^−59^) and 15% (*p = *6×10^−66^), respectively (Table 1). The risk allele also increased BMI by 0.36 kg/m^2^ (∼1 kg in body weight for a person 170 cm tall) (*p = *2×10^−75^), waist circumference by 0.77 cm (*p = *5×10^−43^), and body fat percentage by 0.30% (*p = *2×10^−21^) (Table 1).

**Table 1:** Association of the minor (A−) allele of the rs9939609 SNP or a proxy (*r* ^2^>0.8) in the *FTO* gene with BMI, waist circumference, body fat percentage, risk of obesity, and risk of overweight in a random effects meta-analysis of up to 218,166 adults.

Trait	Geographic Region	*N*	Beta or OR^1^ (95% CI)	*p-*Value	*I* ^2^
**BMI (kg/m^2^)**	All individuals	218,166	0.36 (0.32, 0.40)	1.8×10^−75^	34%
	Europe	164,307	0.32 (0.29, 0.34)	7.6×10^−110^	0%
	North America	47,938	0.42 (0.32, 0.53)	1.4×10^−15^	31%
	Asia	5,921	0.59 (0.33, 0.85)	7.9×10^−6^	39%
**Waist circumference (cm)**	All individuals	159,848	0.77 (0.66, 0.87)	5.4×10^−43^	28%
	Europe	128,811	0.71 (0.60, 0.82)	3.4×10^−37^	20%
	North America	25,117	0.89 (0.60, 1.17)	7.5×10^−10^	17%
	Asia	5,920	1.28 (0.69, 1.86)	1.8×10^−5^	34%
**Body fat percentage (%)**	All individuals	61,509	0.30 (0.24, 0.36)	2.2×10^−21^	1%
	Europe	60,617	0.29 (0.23, 0.36)	2.6×10^−21^	0%
	North America	892	0.79 (0.12, 1.47)	0.021	0%
	Asia	NA	NA	NA	NA
**Risk of obesity (BMI ≥30 versus BMI <25 kg/m^2^)**	All individuals	131,474	1.23 (1.20, 1.26)	7.2×10^−59^	28%
	Europe	97,877	1.22 (1.19, 1.25)	1.7×10^−47^	21%
	North America	29,282	1.26 (1.19, 1.33)	3.3×10^−14^	33%
	Asia	4,315	1.48 (1.25, 1.75)	4.8×10^−6^	0%
**Risk of overweight (BMI ≥25 versus BMI <25 kg/m^2^)**	All individuals	213,564	1.15 (1.13, 1.16)	5.5×10^−66^	10%
	Europe	163,069	1.14 (1.12, 1.16)	5.6×10^−55^	5%
	North America	44,574	1.14 (1.09, 1.18)	2.4×10^−10^	21%
	Asia	5,921	1.26 (1.14, 1.40)	4.9×10^−6^	0%

All models are adjusted for age and sex. Beta is the increase in trait per minor (A−) allele of rs9939609 or a proxy (*r* ^2^>0.8); *I* ^2^ is the heterogeneity between studies in the association of rs9939609 with the trait.

1 Values are beta for all rows except risk of obesity and risk of overweight, for which values are OR.

NA, no data available for analysis.

In children and adolescents, each *FTO* risk allele increased age- and sex-specific BMI by 0.10 *Z*-score units (*p = *1×10^−21^), waist circumference by 0.11 *Z*-score units (*p = *8×10^−16^), and body fat percentage by 0.12 *Z*-score units (*p = *2×10^−11^) (Table S3).

### *FTO*×PA Interaction and Obesity Traits

#### *FTO*×PA interaction and BMI

**Figure 2:**
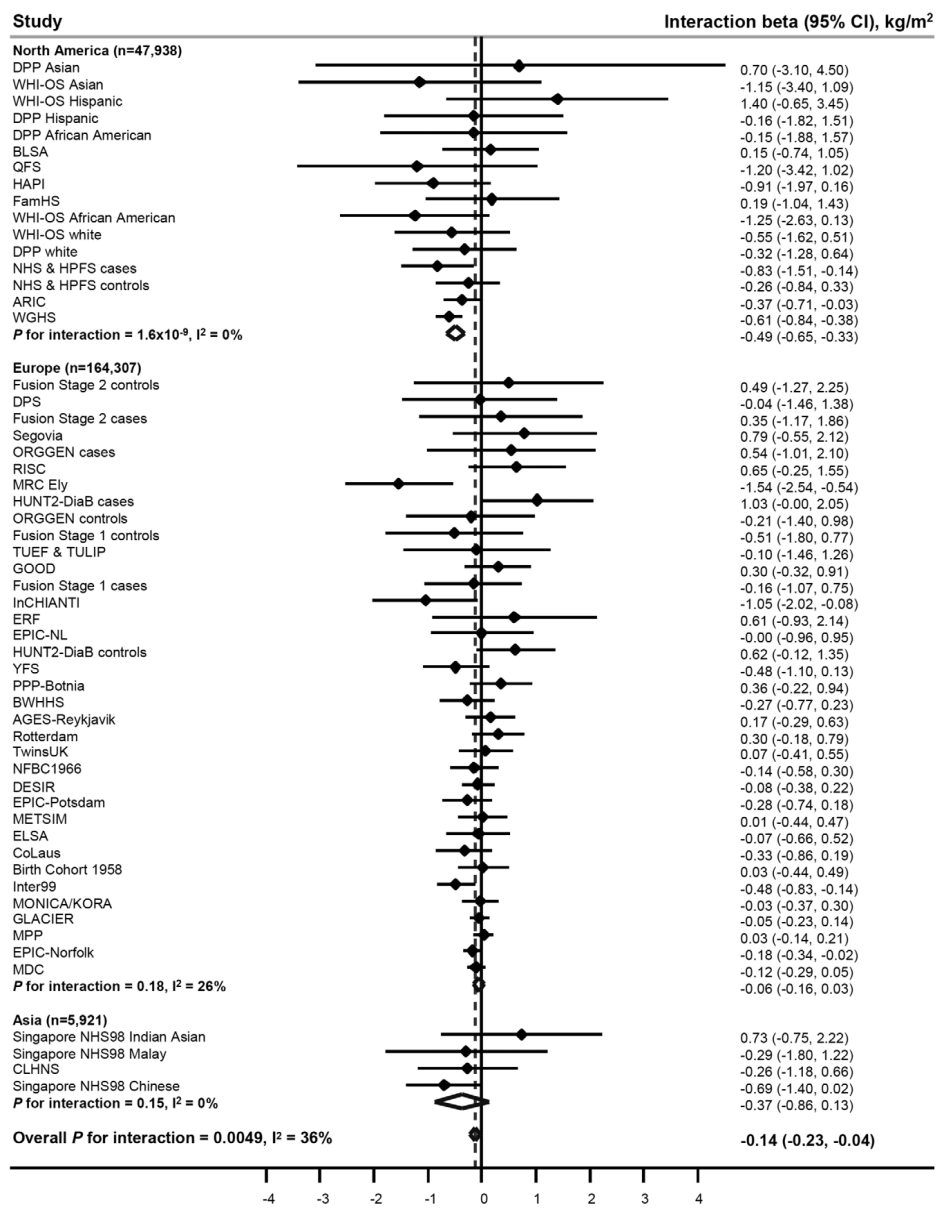
Forest plot of the effect of the interaction between the *FTO* rs9939609 SNP and physical activity on BMI in a random effects meta-analysis of 218,166 adults. The studies are sorted by sample size (largest sample size lowest). Details of the studies are given in Text S1. The interaction beta represents the difference in BMI per minor (A−) allele of rs9939609 comparing physically active individuals to inactive individuals, adjusting for age and sex. For example, a beta_interaction_ of −0.10 kg/m^2^ for BMI represents a 0.10 kg/m^2^ attenuation in the BMI-increasing effect of the rs9939609 minor allele in physically active individuals compared to inactive individuals.

PA significantly (beta_interaction_  =  −0.14 kg/m^2^ per allele, *p*_interaction_  = 0.005) attenuated the association between the *FTO* variant and BMI in our meta-analysis of 218,166 adults (Figure 2; Table 2) (i.e., a beta_interaction_ of −0.14 kg/m^2^ represents the difference in the BMI-increasing effect of the risk allele between physically active and inactive individuals). The magnitude of the effect of the *FTO* risk allele on BMI was 30% smaller in physically active individuals (beta  = 0.32 kg/m^2^) than in inactive individuals (beta  = 0.46 kg/m^2^) (Table 2).

**Table 2:** Effect of the interaction between the rs9939609 SNP or a proxy (*r* ^2^>0.8) and PA on BMI, waist circumference, body fat percentage, risk of obesity, and risk of overweight in a random effects meta-analysis of up to 218,166 adults.

Trait	Geographic Region	Main Effect of rs9939609 in Inactive Individuals			Main Effect of rs9939609 in Active Individuals			Rs9939609×PA Interaction			
		*N*	Beta or OR^1^ (95% CI)	*p-*Value	*N*	Beta or OR^1^ (95% CI)	*p-*Value	*N*	Beta_interaction_ or Interaction OR^1^ (95% CI)	*p-*Value	*I* ^2^
**BMI (kg/m^2^)**	All individuals	54,611	0.46 (0.37, 0.55)	3.7×10^−23^	163,555	0.32 (0.29, 0.36)	4.5×10^−69^	218,166	−0.14 (−0.23, −0.04)	0.0049	36%
	Europe	44,052	0.37 (0.31, 0.44)	1.0×10^−26^	120,255	0.30 (0.27, 0.34)	2.4×10^−62^	164,307	−0.06 (−0.16, 0.03)	0.18	26%
	North America	9,438	0.82 (0.65, 1.00)	2.7×10^−21^	38,500	0.34 (0.25, 0.44)	6.1×10^−12^	47,938	−0.49 (−0.65, −0.33)	1.6×10^−9^	0%
	Asia	1,121	0.78 (0.14, 1.43)	0.017	4,800	0.53 (0.32, 0.75)	1.0×10^−6^	5,921	−0.37 (−0.86, 0.13)	0.15	0%
**Waist circumference (cm)**	All individuals	38,560	1.01 (0.80, 1.22)	2.6×10^−21^	121,288	0.68 (0.58, 0.79)	9.2×10^−35^	159,848	−0.33 (−0.54, −0.12)	0.0018	5%
	Europe	32,519	0.87 (0.65, 1.09)	1.2×10^−14^	96,292	0.65 (0.55, 0.75)	1.4×10^−35^	128,811	−0.22 (−0.44, 0.00)	0.049	4%
	North America	4,921	1.72 (1.16, 2.28)	1.4×10^−9^	20,196	0.65 (0.30, 1.01)	3.2×10^−4^	25,117	−1.02 (−1.60, −0.45)	4.6×10^−4^	0%
	Asia	1,120	1.65 (0.80, 1.22)	0.029	4,800	1.12 (0.61, 1.62)	1.5×10^−5^	5,920	−0.84 (−2.03, 0.35)	0.16	0%
**Body fat percentage (%)**	All individuals	11,839	0.44 (0.30, 0.58)	1.0×101^−9^	49,670	0.28 (0.20, 0.37)	9.4×10^−12^	61,509	−0.19 (−0.35, −0.04)	0.016	0%
	Europe	11,658	0.43 (0.29, 0.57)	3.1×10^−9^	48,959	0.29 (0.20, 0.38)	4.8×10^−10^	60,617	−0.18 (−0.34, −0.03)	0.023	0%
	North America	181	2.03 (0.35, 3.70)	0.018	711	0.48 (−0.26, 1.22)	0.20	892	−1.57 (−3.34, 0.20)	0.082	0%
	Asia	NA	NA	NA	NA	NA	NA	NA	NA	NA	NA
**Risk of obesity (BMI ≥30 versus BMI <25 kg/m^2^)**	All individuals	32,774	1.30 (1.24, 1.36)	1.1×10^−29^	97,779	1.22 (1.19, 1.25)	1.0×10^−46^	131,474	0.92 (0.88, 0.97)	0.0010	5%
	Europe	26,139	1.27 (1.22, 1.33)	2.9×10^−29^	71,738	1.21 (1.17, 1.25)	9.1×10^−35^	97,877	0.94 (0.90, 0.99)	0.028	0%
	North America	5,777	1.43 (1.28, 1.60)	6.0×10^−10^	23,505	1.22 (1.15, 1.30)	1.0×10^−9^	29,282	0.85 (0.75, 0.98)	0.024	24%
	Asia	858	1.86 (1.17, 2.93)	0.0082	3,457	1.41 (1.18, 1.70)	1.9×10^−4^	4,315	0.74 (0.46, 1.20)	0.23	0%
**Risk of overweight (BMI ≥25 versus BMI <25 kg/m^2^)**	All individuals	53,726	1.19 (1.15, 1.23)	2.0×10^−22^	159,838	1.14 (1.12, 1.16)	2.1×10^−42^	213,564	0.95 (0.91, 0.99)	0.015	20%
	Europe	43,833	1.17 (1.13, 1.20)	5.8×10^−26^	119,236	1.14 (1.11, 1.16)	3.2×10^−29^	163,069	0.96 (0.92, 1.01)	0.090	14%
	North America	8,772	1.24 (1.11, 1.39)	1.8×10^−4^	35,802	1.13 (1.09, 1.17)	1.0×10^−12^	44,574	0.89 (0.80, 0.99)	0.034	21%
	Asia	1,121	1.21 (0.79, 1.87)	0.38	4,800	1.26 (1.13, 1.41)	4.9×10^−5^	5,921	0.99 (0.67, 1.48)	0.98	50%

All models are adjusted for age and sex. Beta is the increase in trait per minor allele of rs9939609 or a proxy (*r* ^2^>0.8); beta_interaction_ is the difference in trait per minor allele of rs9939069 comparing physically active individuals to inactive individuals, e.g., a beta_interaction_ of −0.14 kg/m^2^ for BMI represents a 0.14 kg/m^2^ attenuation in the BMI-increasing effect of the rs9939609 minor allele in physically active individuals compared to inactive individuals; *I* ^2^ is the heterogeneity between studies in the meta-analysis; interaction OR is the ratio of ORs (OR[physically active]/OR[inactive]) per minor allele of rs9939609, e.g., an interaction OR of 0.92 for risk of obesity indicates that the obesity-increasing effect of the rs9939609 minor allele in physically active individuals is 0.92 of the effect in inactive individuals.

1 Values are beta/beta_interaction_ for all rows except risk of obesity and risk of overweight, for which values are OR/interaction OR.

NA, no data available for analysis.

To examine the sources of heterogeneity between studies, which was moderate (*I* ^2^ = 36%) (Table 2; Figure 2), we used meta-regression. The meta-regression indicated heterogeneity by geographic region (North America versus Europe) in the interaction (*p = *0.001) (Tables S4 and S5). When we subsequently stratified our meta-analysis by geographic region, the attenuating effect of PA on the association between the *FTO* variant and BMI was more pronounced in North American populations than in European populations (*p*_difference_  = 5×10^−6^) (Table 2; Figure 2). More specifically, the BMI-increasing effect of the *FTO* risk allele in physically active North Americans was 59% smaller than in inactive North Americans (beta  = 0.34 versus 0.82 kg/m^2^, respectively), whereas the attenuation in the BMI-increasing effect of the risk allele in physically active Europeans compared with inactive Europeans was only 19% (beta  = 0.30 versus 0.37 kg/m^2^, respectively) (Table 2). There was no heterogeneity among North American studies (*I* ^2^ = 0%), whereas moderate heterogeneity was observed among European studies (*I* ^2^ = 26%) (Table 2; Figure 2). In a further sub-group meta-regression, none of the covariates explained a significant proportion of the remaining heterogeneity observed in Europeans.

To test for the presence of “positive results bias” (i.e., whether studies with positive results were more likely to participate in our meta-analysis than those with negative or inconclusive results), we drew a funnel plot of the interaction beta coefficients and standard errors and conducted Begg and Egger tests for bias. The funnel plot was symmetrical, and the results for Begg and Egger tests were non-significant (*p = *0.9 and *p = *0.8, respectively), indicating that our results were not affected by positive results bias (Figure S1).

**Figure 3:**
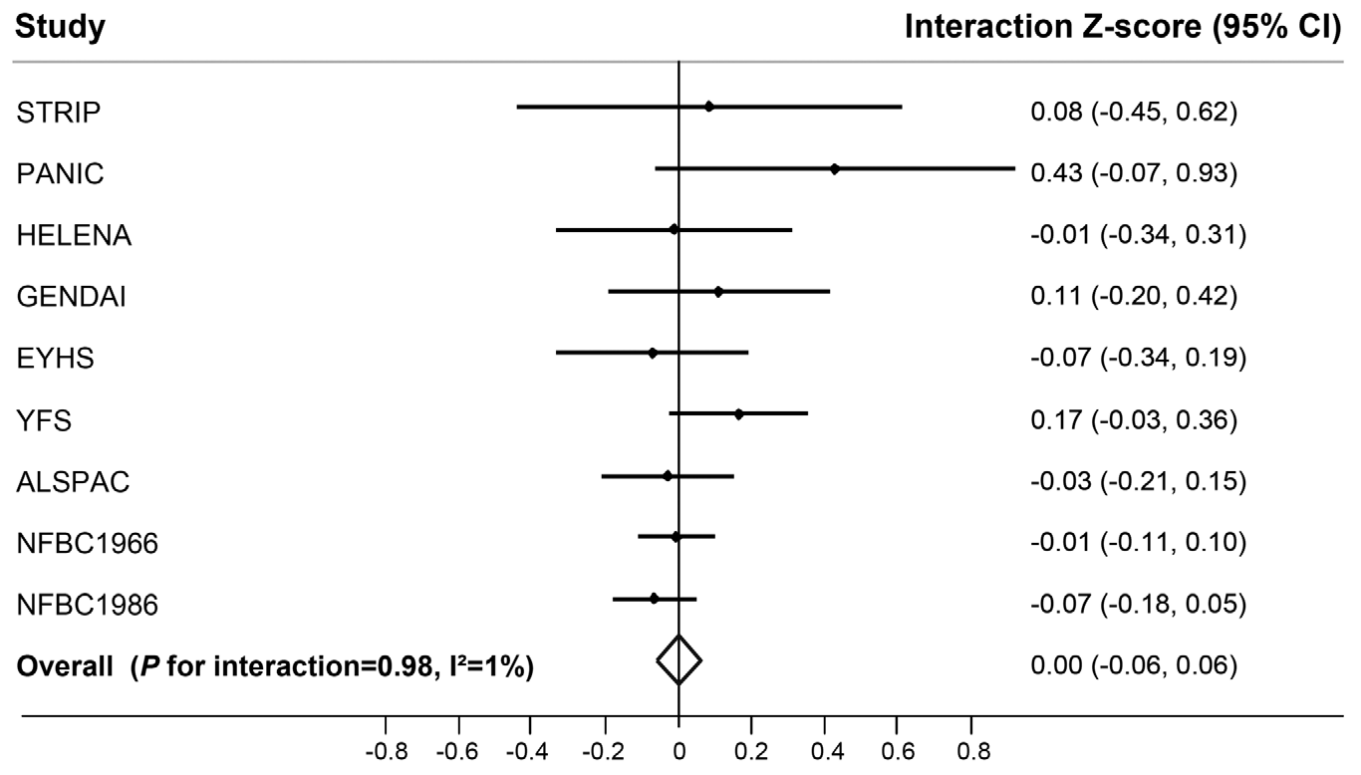
Forest plot of the effect of the interaction between the *FTO* rs9939609 SNP and physical activity on BMI in a random effects meta-analysis of 19,268 children and adolescents. The studies are sorted by sample size (largest sample size lowest). Details of the studies are given in Text S1. The interaction *Z*-score represents the difference in age- and sex-standardized BMI per minor (A−) allele of rs9939609 comparing physically active children to inactive children. For example, a beta_interaction_ of −0.1 represents a 0.1 unit attenuation in the BMI *Z*-score-increasing effect of the rs9939609 minor allele in physically active children compared to inactive children.

While we observed a strong effect of the *FTO* risk allele on BMI in children and adolescents (Table S3), this effect was not modified by their PA level (*p*_interaction_  = 0.98) (Figure 3). There was no heterogeneity between the studies (*I* ^2^ = 1%).

#### *FTO*×PA interaction and risk of obesity and overweight

Consistent with the meta-analysis of BMI, PA attenuated the effect of the *FTO* risk allele on the odds of obesity (*p*_interaction_  = 0.001) and on the odds of overweight (*p*_interaction_  = 0.02) (Table 2; Figures S2 and S3). The odds of obesity for the *FTO* risk allele were 27% smaller (odds ratio [OR] = 1.22 versus 1.30, respectively) and the odds of overweight were 26% smaller (OR = 1.14 versus 1.19, respectively) in physically active individuals than in inactive individuals (Table 2). Similar to the results for BMI, there seemed to be a more pronounced *FTO*×PA interaction effect in North American populations than in Europeans: the risk-attenuating effect of PA was more than double in North Americans compared to Europeans (Table 2; Figures S2 and S3). The differences in the interaction effect between North Americans and Europeans on the odds of obesity and overweight were, however, not significant (*p*_difference_  = 0.2 for both obesity and overweight).

#### *FTO*×PA interaction on waist circumference and body fat percentage

We observed a significant effect of the *FTO*×PA interaction on waist circumference (beta_interaction_  = −0.33 cm, *p*_interaction_  = 0.002) and body fat percentage (beta_interaction_  = −0.19%, *p*_interaction_  =  0.02) (Table 2; Figures S4 and S5). The influence of the *FTO* risk allele on waist circumference was 33% smaller and the influence on body fat percentage 36% smaller in physically active individuals than in inactive individuals (Table 2). Similar to the results for BMI, the effect of the *FTO*×PA interaction on waist circumference was also more pronounced in North American populations (beta_interaction_  =  −1.02 cm) than in Europeans (beta_interaction_  =  −0.22 cm) (*p*_difference_  =  0.01) (Table 2; Figure S4). We found a similar difference for body fat percentage (beta_interaction_  =  −1.57% in North Americans versus beta_interaction_  =  −0.18% in Europeans) that, however, did not reach significance (*p*_difference_  = 0.1), but few North American individuals had data on body fat percentage (*n = *892) (Table 2; Figure S5).

The effect of the *FTO* risk allele on waist circumference and body fat percentage in children and adolescents was not modified by their PA level (*p*_interaction_  = 0.7 and 0.4, respectively) (Figures S6 and S7). There was no heterogeneity between the studies in these meta-analyses (*I* ^2^  =  0%).

### Association of *FTO* with Physical Activity

There was no association between *FTO* and the level of PA in adults (*p = *0.2) (Figure S8) or children (*p = *0.6) (Figure S9). No between-study heterogeneity was found in these meta-analyses either (*I* ^2^≤1%).

## Discussion

By combining data from 218,166 adults from 45 studies, we confirm that PA attenuates the influence of *FTO* variation on BMI and obesity. The association of the *FTO* rs9939609 variant with BMI and with the odds of obesity was reduced by approximately 30% in physically active compared to inactive adults. We also found an interaction effect on the odds of overweight and on waist circumference and body fat percentage. No interaction between *FTO* and PA was found in our meta-analysis of 19,268 children and adolescents.

Our findings are highly relevant for public health. They emphasize that PA is a particularly effective way of controlling body weight in individuals with a genetic predisposition towards obesity and thus contrast with the determinist view held by many that genetic influences are unmodifiable. While our findings carry an important public health message for the population in general, they do not have an immediate impact at the individual level. More specifically, targeting PA interventions based on *FTO* genotype screening only would not accurately identify those who would benefit most of such intervention, as the effect of the *FTO* variant on body weight is relatively small (∼1 kg) and the attenuation of this effect by PA is limited. Of interest is that current evidence does not suggest that genetic testing would lead to an increased motivation of individuals to improve their lifestyle [33]. On the contrary, a recent study suggests that those shown to be genetically susceptible to obesity may even worsen their dietary habits [33]. Convincing evidence of gene–lifestyle interactions, however, might give people a sense of control that risk-reducing behaviors can be effective in prevention. Thus, identifying interactions between genes and lifestyle is important as it demonstrates that a genetic susceptibility to obesity is modifiable by lifestyle. Furthermore, insights from gene–lifestyle interactions contribute to elucidating the mechanisms behind genetic regulation of obesity, which may help in the development of new treatments in the future.

Interestingly, we found a geographic difference in the interaction of *FTO* with PA, which was consistent across the studied phenotypes. In particular, the interaction was stronger in North American populations than in populations from Europe. Reasons for the observed geographic difference are unclear. As the participating North American and European studies are mainly representative of individuals of European descent, genetic differences between them are small and unlikely to substantially contribute to the observed difference in the interaction. However, we speculate that the geographic difference may, at least in part, be related to the lower average levels of PA in individuals living in North America than in Europe [34],[35]. The *FTO*×PA interaction effect may materialize more in populations with a high prevalence of very sedentary individuals [17]. Furthermore, sedentariness may also associate with other lifestyle factors that may contribute to the interaction, such as unhealthy diet [16],[19],[20], which we were not able to adjust for in our meta-analyses and which may be more prevalent in North American populations than in Europeans [36]. Finally, there were differences in the measurement methods used to assess PA between North American and European studies. More specifically, all North American studies quantified PA using a continuous PA variable, whereas many European studies used categorical variables. As a result, the overall number of individuals defined as inactive was smaller in North American (20%) than in European studies (27%). We also found that PA was associated with a 1.34 kg/m^2^ lower BMI in North American populations, but with only a 0.72 kg/m^2^ lower BMI in Europeans. As misclassification in exposure measurements usually biases the effect towards the null, it is possible that lower accuracy of PA measurements in European studies may have deflated the effect of PA on BMI, as well as the interaction between *FTO* and PA. In our meta-regressions, however, we did not find a significant association between the measurement of PA and the observed *FTO*×PA interaction effect (Tables S4 and S5). Nevertheless, it is likely that our overall effect estimate for the interaction is a considerable underestimate of the true effect because of measurement error of PA.

In studies with continuous measures of PA, we chose to use a definition of “inactivity” based on a relative (lowest 20%) cut-off of PA levels. The use of a cut-off based on fixed percentage may have introduced heterogeneity as the percentage may correspond to different absolute PA values in the participating studies. However, the use of an absolute cut-off might have led to even greater heterogeneity, because of the wide differences in the measurement instruments that were used to provide the continuous measures of PA. In theory, the accuracy of a relative PA cut-off could be improved by choosing a specific cut-off for each country on the basis of national PA data. In practice, however, comparing PA data between countries is difficult, as prevalence estimates for sedentariness have been assessed by different survey instruments, which sometimes have also changed over time, and prevalence estimates are not always available from representative samples of the population.

The present meta-analysis was based on cross-sectional data and thus does not provide information on the longitudinal relationships between variables. While germline DNA remains stable throughout the life course, PA levels may change and may be confounded by other lifestyle and environmental factors that correlate with PA and body weight. So far, only three prospective follow-up studies (*n*_range_  = 502 to 15,844) on the interaction between *FTO* and PA have been reported, and none showed an interaction between *FTO* and baseline PA, or change in PA, on weight change during follow-up [21]–[23]. Although studies investigating PA alone did not find an interaction, the Diabetes Prevention Program in the US showed an interaction between *FTO* and a 1-y lifestyle intervention, consisting of PA, diet, and weight loss combined, on change in subcutaneous fat area among 869 individuals [37]. The minor allele of the *FTO* variant was associated with an increase in subcutaneous fat area in the control group but not in the lifestyle intervention group [37]. Two studies have tested whether *FTO* modified the effect of a standardized exercise program on change in body weight in individuals who were sedentary at baseline, but results were inconsistent. While the first study showed a greater weight loss for the carriers of the major (C−) allele of *FTO* rs8050136 after a 20-wk endurance training program among 481 men and women [38], a subsequent study with a 6-mo endurance training program among 234 women indicated weight loss benefits for the carriers of the minor (A−) allele of the same variant [39]. These studies may have been insufficiently powered to detect an interaction between *FTO* variation and exercise intervention. A meta-analysis of prospective studies may be required to confirm or refute whether there is an interaction between changes in PA and *FTO* on weight gain in a sufficiently powered population sample. Finally, a large-scale randomized controlled trial would be needed to infer causality for the interaction between PA and *FTO*.

We found no interaction between the *FTO* variant and PA on BMI in children and adolescents, which could be because of low statistical power, as the sample size was 11 times smaller than in the meta-analysis of adults. Even so, the effect size of the interaction was null, suggesting that no attenuation of PA on the BMI-increasing effect of *FTO* would be found, even if a larger sample was meta-analyzed. The lack of interaction in children may, at least in part, be due to the weak association between PA and childhood BMI and the higher activity levels in children than in adults [30]. Despite the fact that BMI is a noninvasive and easy-to-obtain measure of adiposity, its weakness is that it does not distinguish lean body mass from fat mass and may therefore not be the best measure of adiposity in children. Indeed, the associations of PA with waist circumference and body fat percentage were significant, and the effect of the *FTO*×PA interaction on body fat percentage pointed towards a slightly decreased effect of the *FTO* risk allele in physically active children as compared to sedentary children.

We designed a meta-analysis based on a de novo analysis of data according to a standardized plan in all studies identified as having available data. The analytical consistency across studies, which helped minimize between-study heterogeneity, and the pooling of all identified data, which minimized biases related to study selection, are major strengths of our meta-analysis. A greater consistency and statistical power could ultimately be reached only through the establishment of large single or multicenter studies using standardized methods and precise measurement of PA.

In summary, we have established that PA attenuates the association of the *FTO* gene with adult BMI and obesity by approximately 30%. We have also demonstrated that large-scale international collaborations are useful for confirming interactions between genes and lifestyle.

## Supporting Information

Figure S1 Funnel plot of the effect of the interaction between the *FTO* rs9939609 SNP and physical activity on BMI in a random effects meta-analysis of 45 studies (218,166 adults).
(PDF)

Figure S2 Forest plot of the effect of the interaction between the *FTO* rs9939609 SNP and physical activity on risk of obesity (BMI ≥30 versus BMI <25 kg/m^2^) in a random effects meta-analysis of 131,474 adults.
(PDF)

Figure S3 Forest plot of the effect of the interaction between the *FTO* rs9939609 SNP and physical activity on risk of overweight (BMI ≥25 versus BMI <25 kg/m^2^) in a random effects meta-analysis of 213,564 adults.
(PDF)

Figure S4 Forest plot of the effect of the interaction between the *FTO* rs9939609 SNP and physical activity on waist circumference in a random effects meta-analysis of 159,848 adults.
(PDF)

Figure S5 Forest plot of the effect of the interaction between the *FTO* rs9939609 SNP and physical activity on body fat percentage in a random effects meta-analysis of 61,509 adults.
(PDF)

Figure S6 Forest plot of the effect of the interaction between the *FTO* rs9939609 SNP and physical activity on age- and sex-standardized waist circumference in a random effects meta-analysis of 12,392 children and adolescents.
(PDF)

Figure S7 Forest plot of the effect of the interaction between the *FTO* rs9939609 SNP and physical activity on age- and sex-standardized body fat percentage in a random effects meta-analysis of 6,864 children and adolescents.
(PDF)

Figure S8 Forest plot of the association of the *FTO* rs9939609 SNP with physical activity in a random effects meta-analysis of 218,166 adults.
(PDF)

Figure S9 Forest plot of the association of the *FTO* rs9939609 SNP with physical activity in a random effects meta-analysis of 19,268 children and adolescents.
(PDF)

Table S1 Association of physical activity with BMI, waist circumference, body fat percentage, risk of obesity, and risk of overweight in a random effects meta-analysis of up to 218,166 adults.
(PDF)

Table S2 Association of physical activity with age- and sex-standardized BMI, waist circumference, and body fat percentage in a random effects meta-analysis of up to 19,268 children and adolescents.
(PDF)

Table S3 Association of the minor (A−) allele of the *FTO* rs9939609 SNP with age- and sex-standardized BMI, waist circumference, and body fat percentage in a random effects meta-analysis of up to 19,268 children and adolescents.
(PDF)

Table S4 Results of meta-regression showing the associations of all study characteristics combined with the *FTO*×PA interaction effect on BMI in adults.
(PDF)

Table S5 Results of meta-regressions for the association of each study characteristic separately with the *FTO*×PA interaction effect on BMI in adults.
(PDF)

Text S1 Supplementary descriptive information about the studies included in the meta-analyses.
(PDF)

Text S2 Acknowledgments and funding.
(PDF)

## Abbreviations

BMI: body mass index
OR: odds ratio
PA: physical activity
SNP: single nucleotide polymorphism
