# Supplementary figures and images for: Physical Activity Attenuates the Influence of FTO Variants on Obesity Risk: A Meta-Analysis of 218,166 Adults and 19,268 Children
Source: PLoS Med. 2011 Nov 1;8(11):e1001116. doi: 10.1371/journal.pmed.1001116 (PMC3206047; doi:10.1371/journal.pmed.1001116)

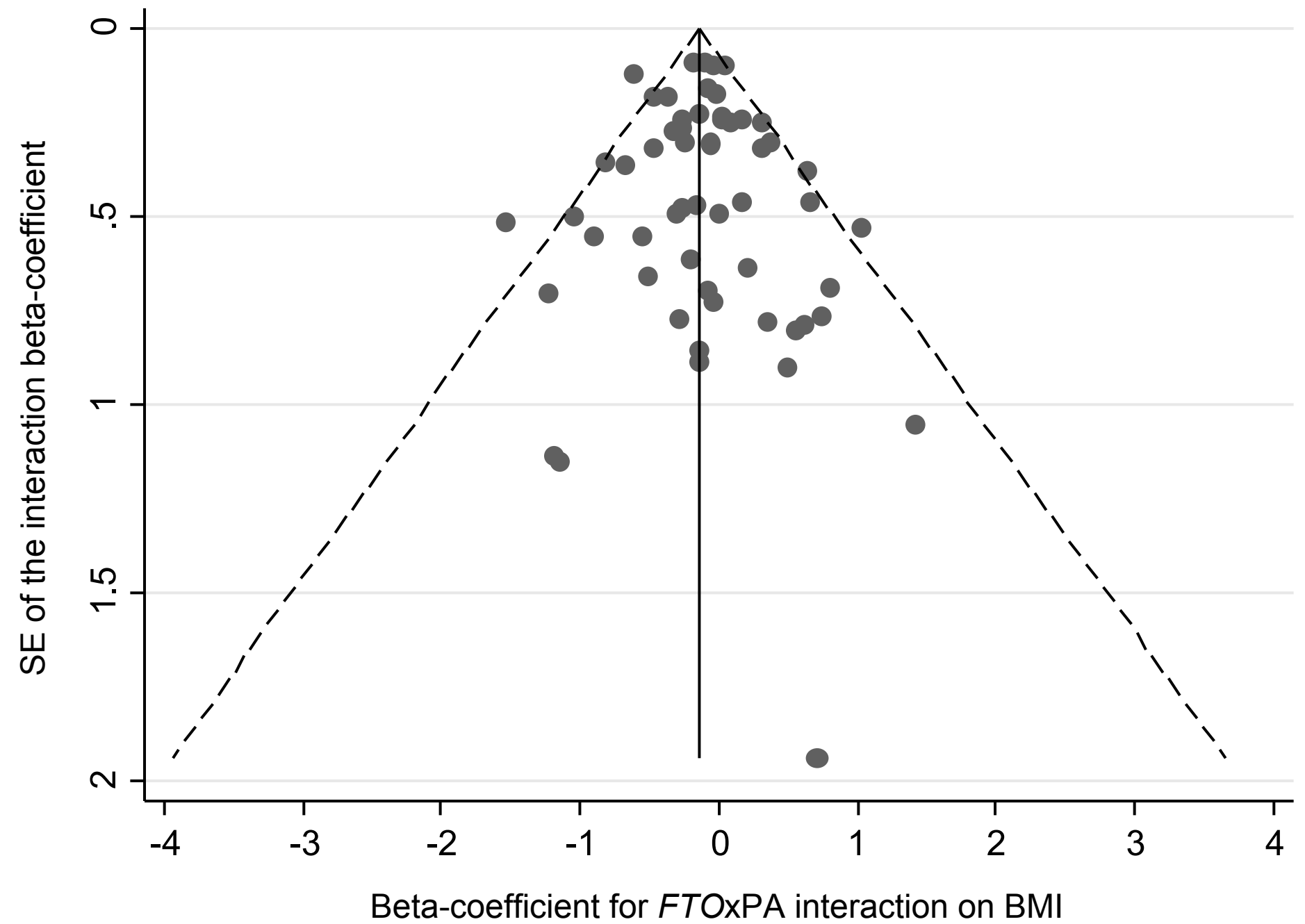

Supplement: Figure S1 — Funnel plot of the effect of the interaction between the FTO rs9939609 SNP and physical activity on BMI in a random effects meta-analysis of 45 studies (218,166 adults). (PDF) [file pmed.1001116.s001.pdf]

## Study

## Interaction-OR (95% CI)

## North America (n=29,282)

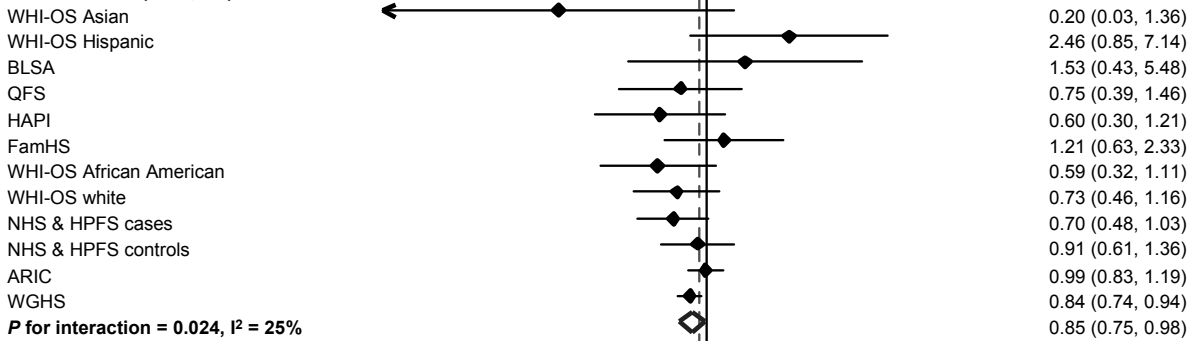

## Europe (n=97,877)

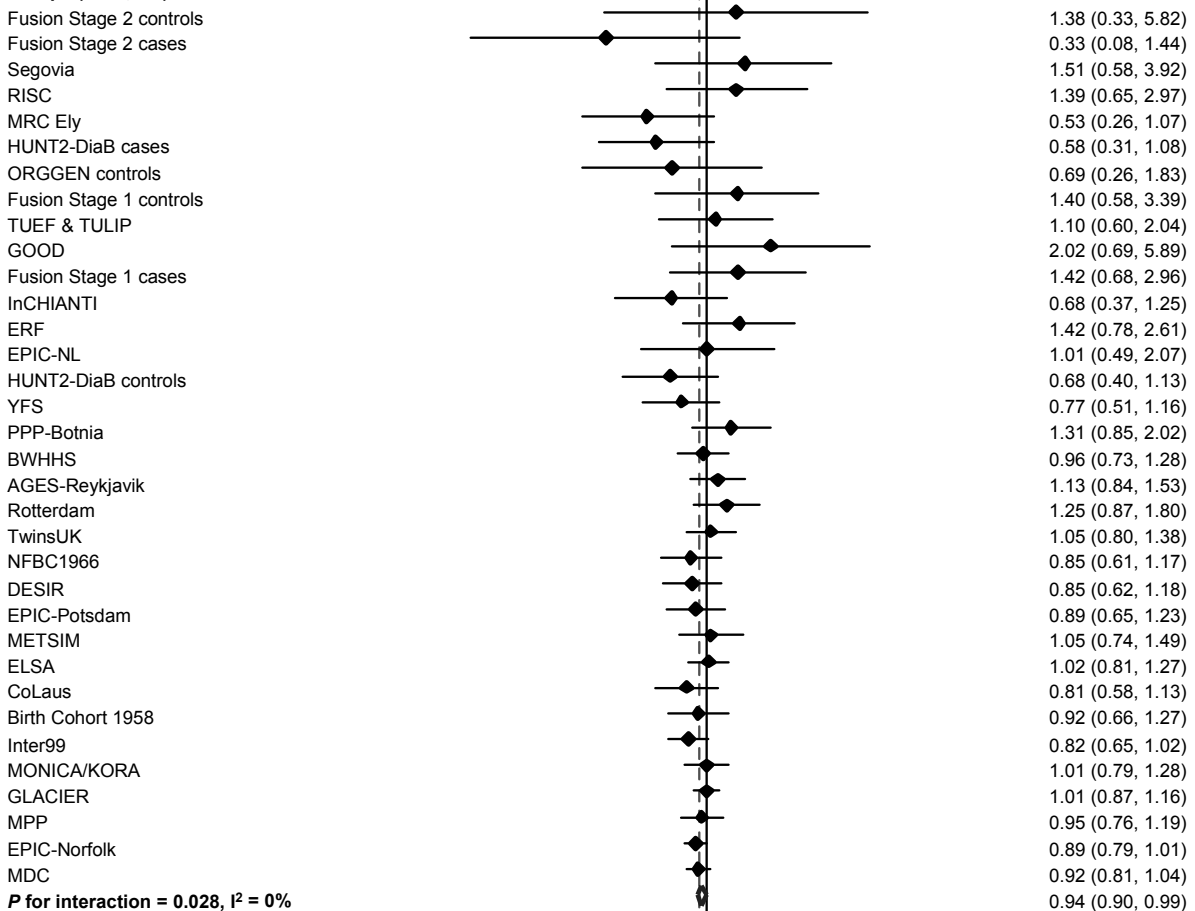

## Asia (n=4,315)

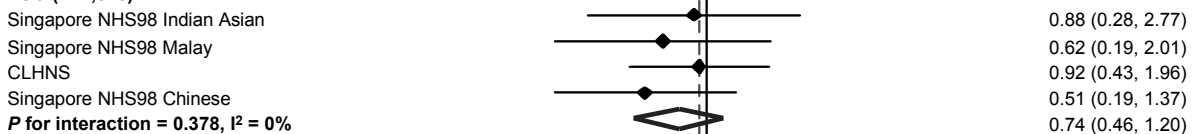

**P for interaction = 0.0010, I<sup>2</sup> = 5%**

**0.92 (0.88, 0.97)**

0.063 0.125 0.25 0.5 1 2 4 8 16

Supplement: Figure S2 — Forest plot of the effect of the interaction between the FTO rs9939609 SNP and physical activity on risk of obesity (BMI ≥30 versus BMI <25 kg/m2) in a random effects meta-analysis of 131,474 adults. (PDF) [file pmed.1001116.s002.pdf]

## Study

## Interaction-OR (95% CI)

**North America (n=44,574)**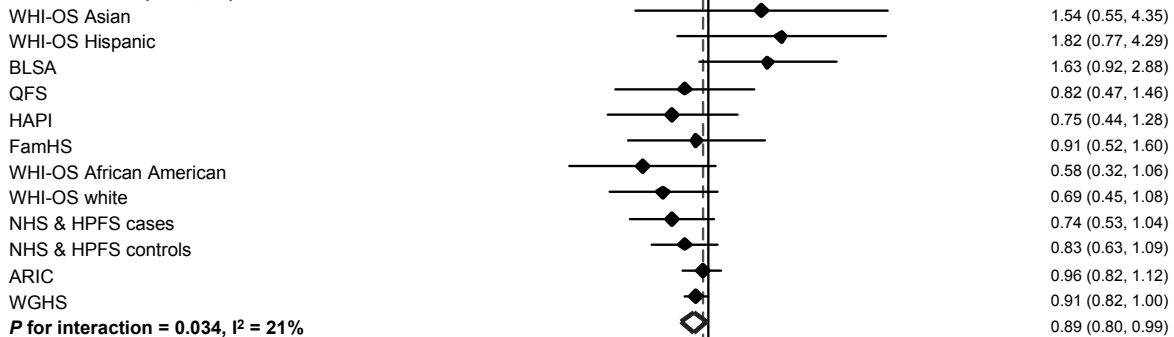**Europe (n=163,069)**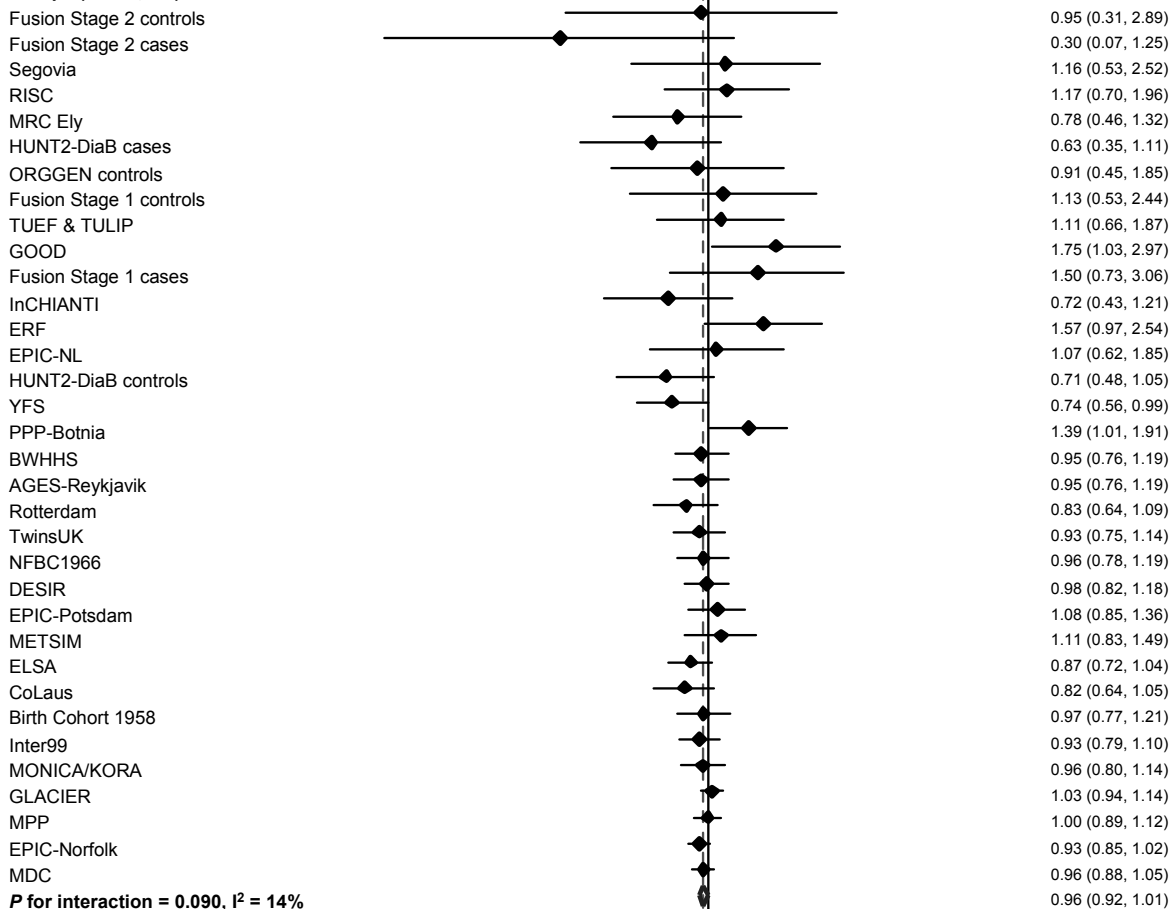**Asia (n=5,921)**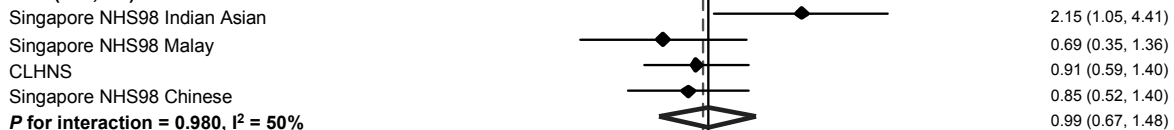**P for interaction = 0.015, I<sup>2</sup> = 20%****0.95 (0.91, 0.99)**

0.063 0.125 0.25 0.5 1 2 4 8 16

Supplement: Figure S3 — Forest plot of the effect of the interaction between the FTO rs9939609 SNP and physical activity on risk of overweight (BMI ≥25 versus BMI <25 kg/m2) in a random effects meta-analysis of 213,564 adults. (PDF) [file pmed.1001116.s003.pdf]

## Study

## Interaction beta (95% CI), cm

**North America (25,117)**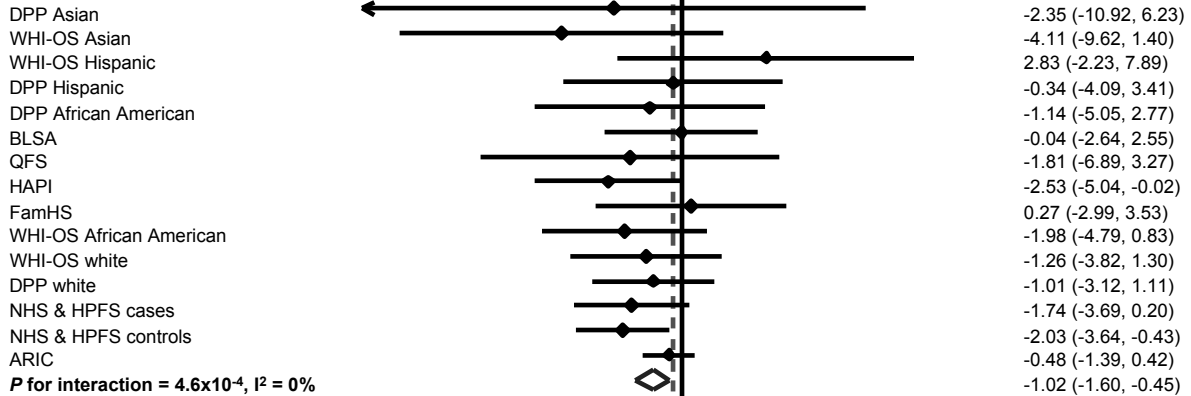**Europe (n=128,811)**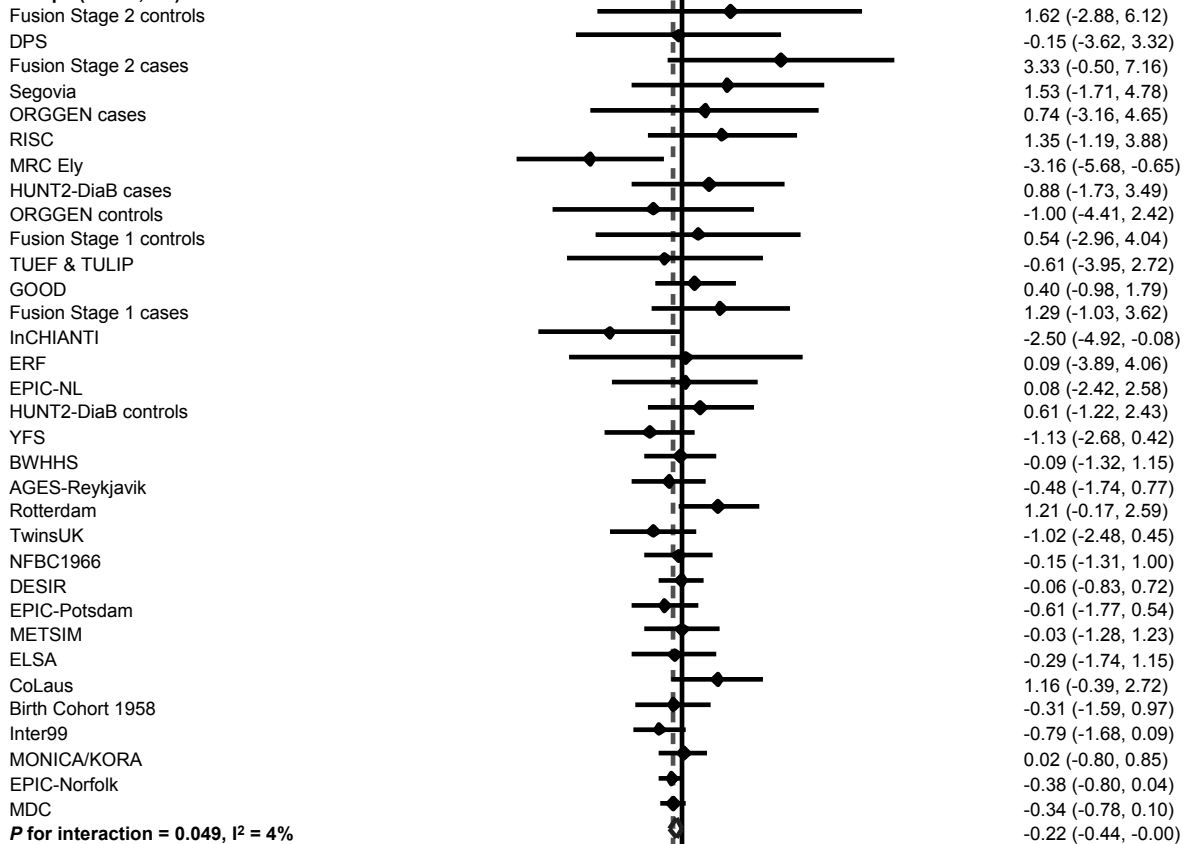**Asia (n=5,920)**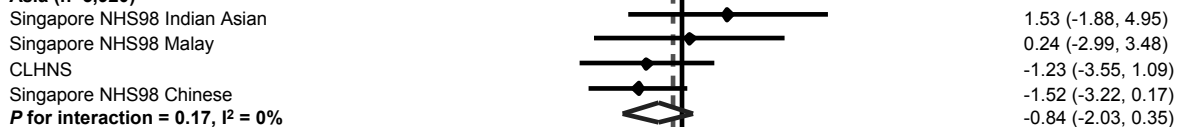**P for interaction = 0.0018,  $I^2 = 5\%$** **-0.33 (-0.54, -0.12)**

-8 -6 -4 -2 0 2 4 6 8

Supplement: Figure S4 — Forest plot of the effect of the interaction between the FTO rs9939609 SNP and physical activity on waist circumference in a random effects meta-analysis of 159,848 adults. (PDF) [file pmed.1001116.s004.pdf]

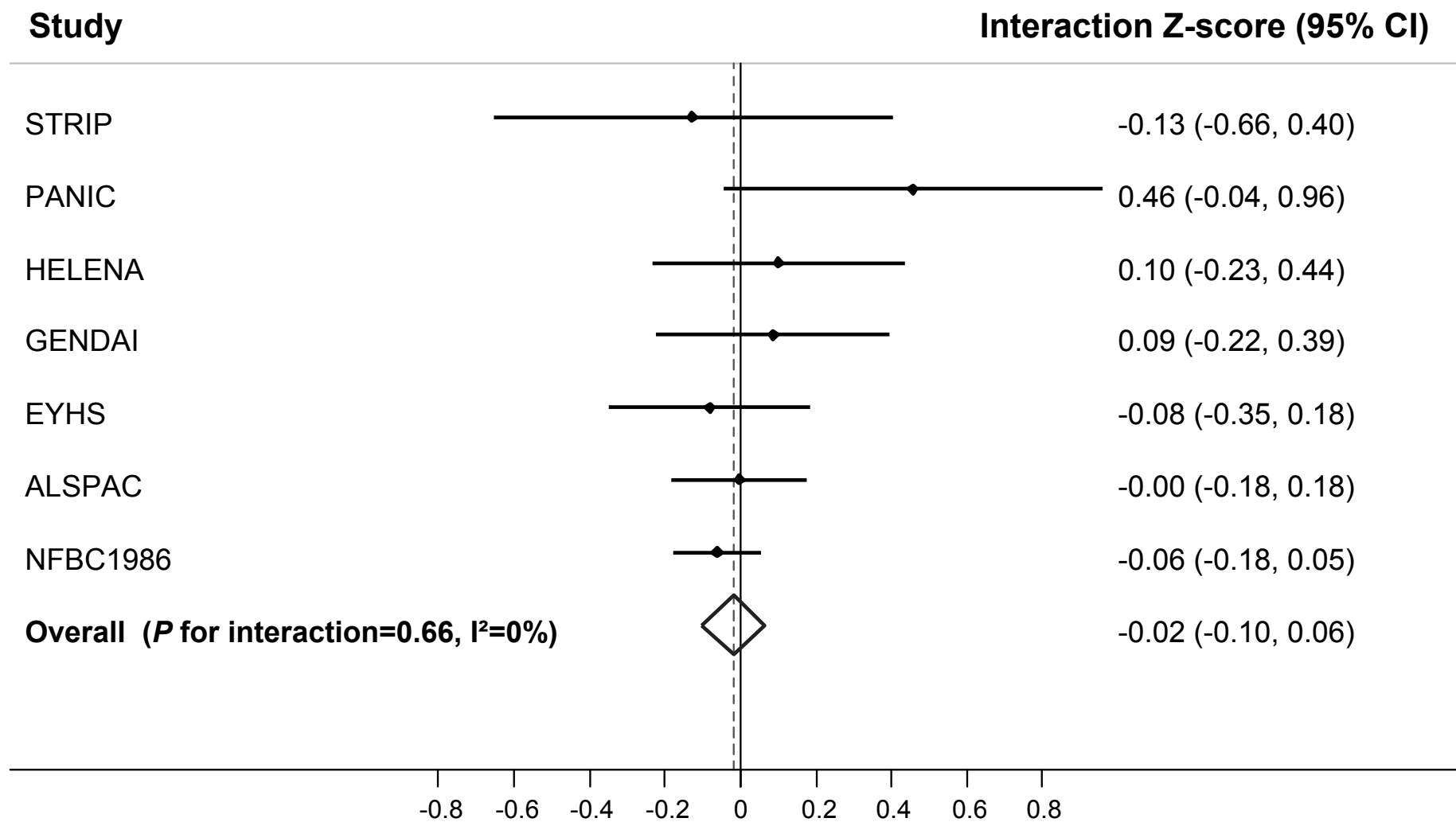

Supplement: Figure S6 — Forest plot of the effect of the interaction between the FTO rs9939609 SNP and physical activity on age- and sex-standardized waist circumference in a random effects meta-analysis of 12,392 children and adolescents. (PDF) [file pmed.1001116.s006.pdf]

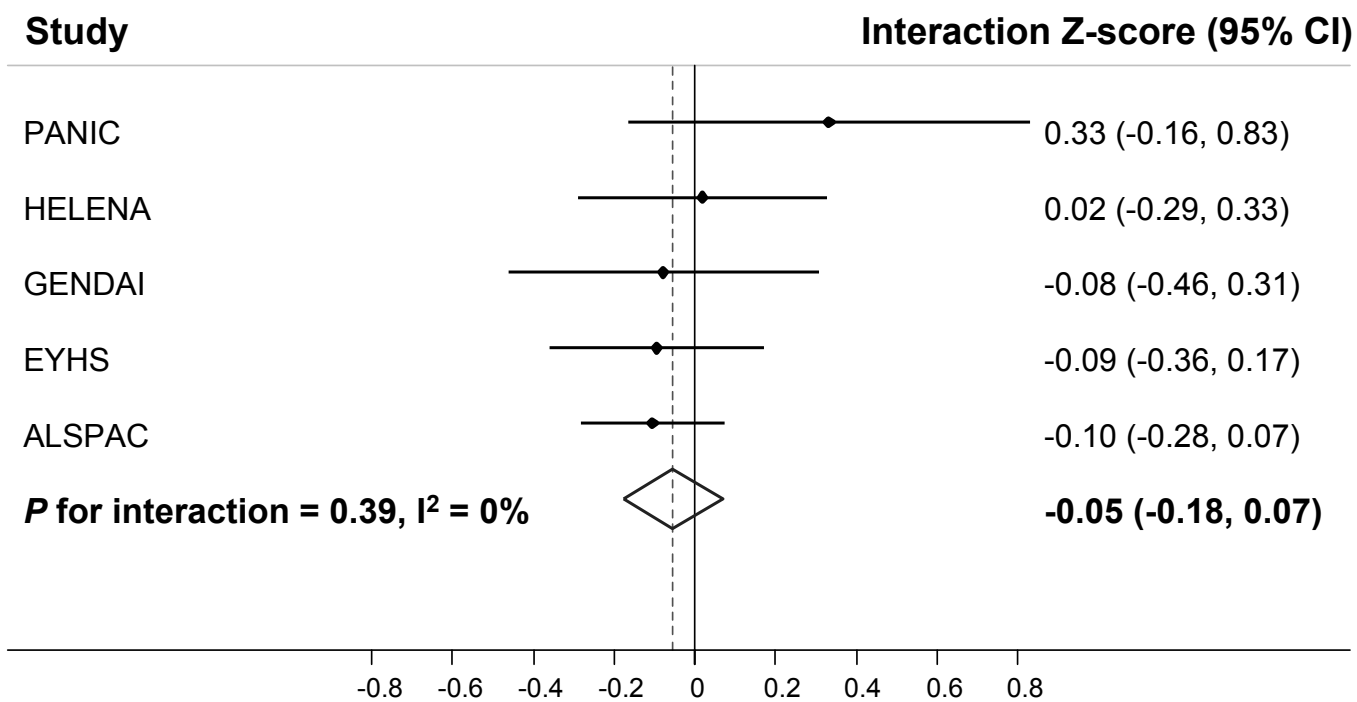

Supplement: Figure S7 — Forest plot of the effect of the interaction between the FTO rs9939609 SNP and physical activity on age- and sex-standardized body fat percentage in a random effects meta-analysis of 6,864 children and adolescents. (PDF) [file pmed.1001116.s007.pdf]

**North America (n=47,938)**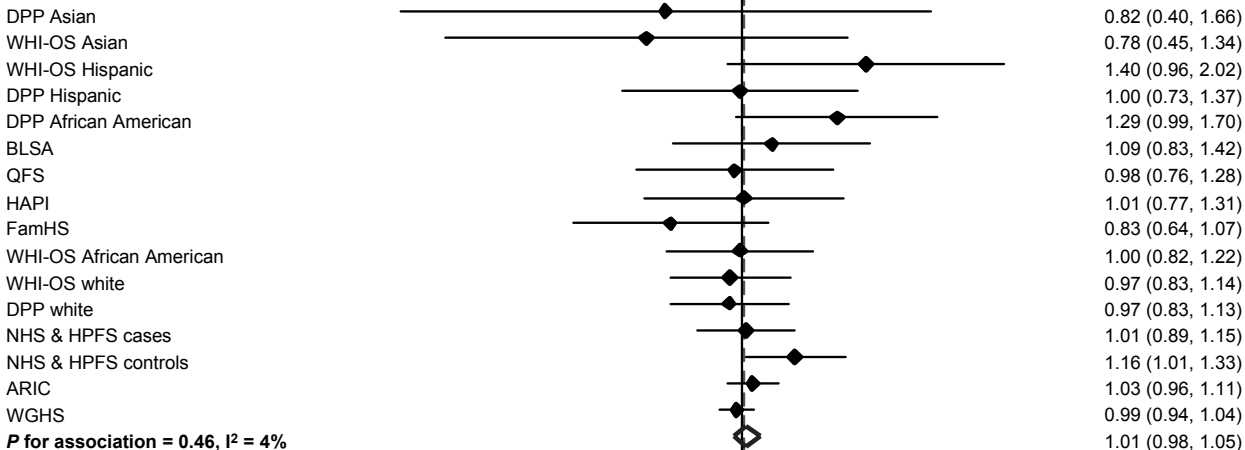**Europe (n=164,307)**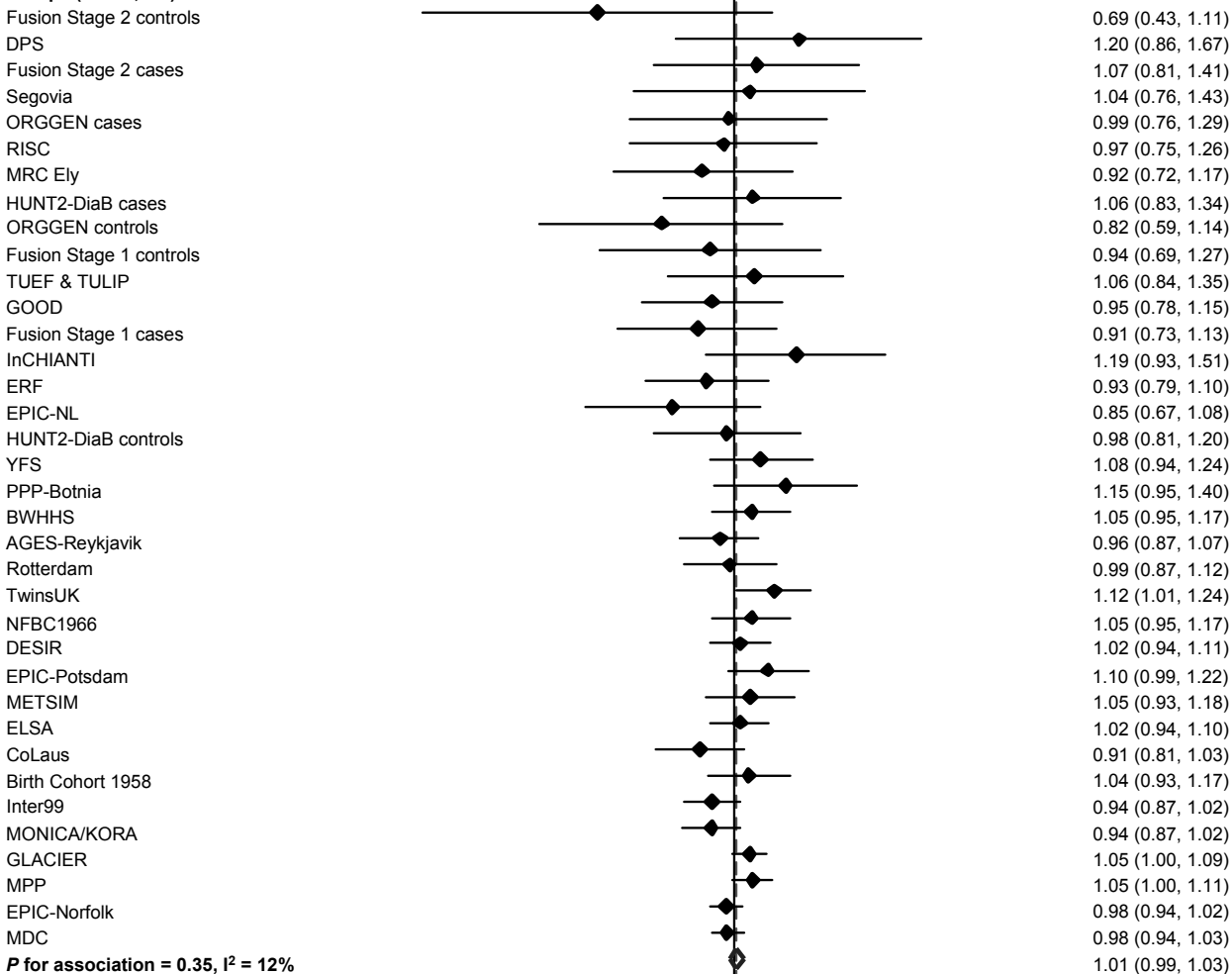**Asia (n=5,921)**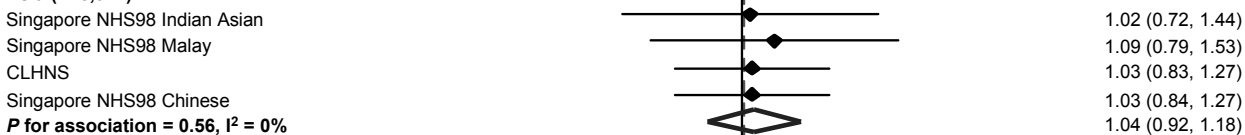**Overall P for association = 0.20, I<sup>2</sup> = 1%****1.01 (0.99, 1.03)**

0.5 0.6 0.7 0.8 0.9 1 1.2 1.4 1.6 1.8 2.0

Supplement: Figure S8 — Forest plot of the association of the FTO rs9939609 SNP with physical activity in a random effects meta-analysis of 218,166 adults. (PDF) [file pmed.1001116.s008.pdf]

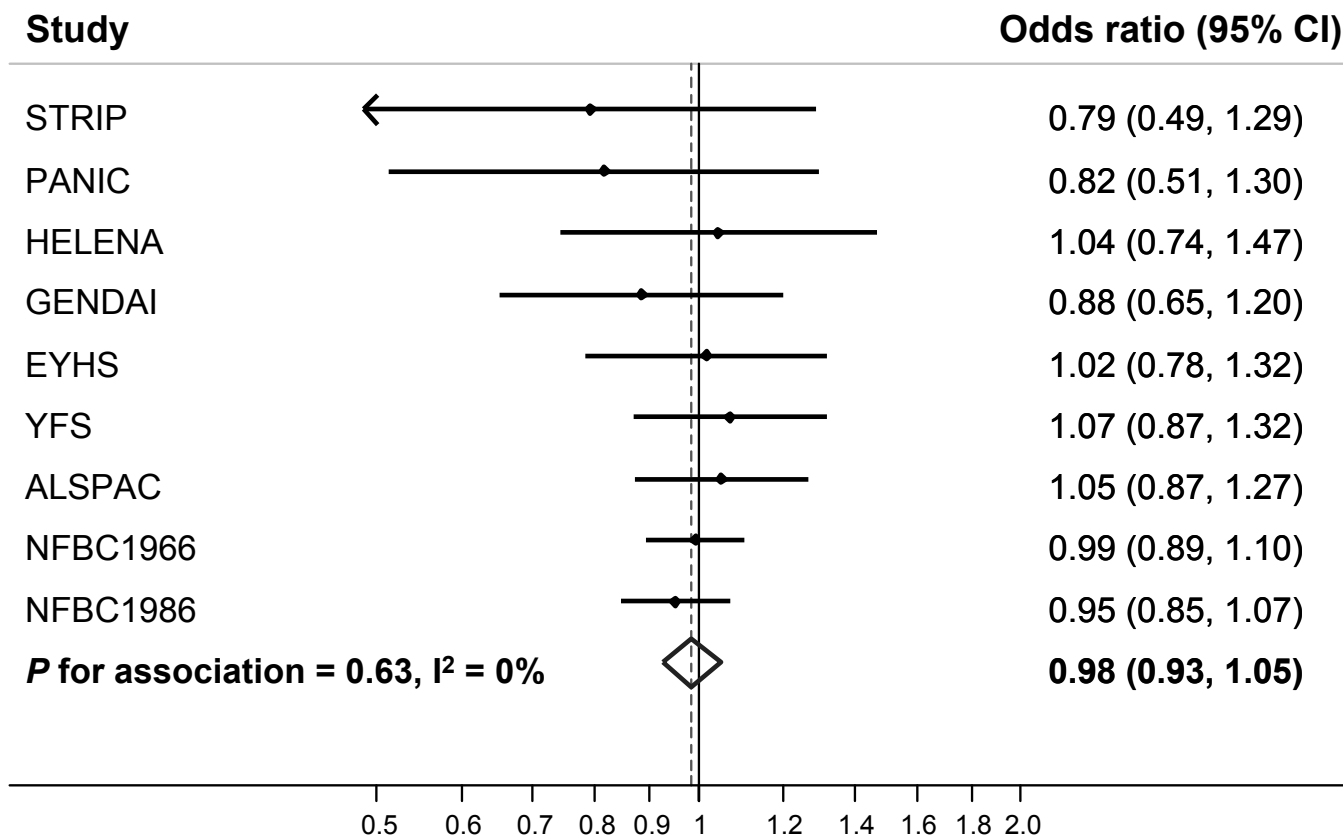

Supplement: Figure S9 — Forest plot of the association of the FTO rs9939609 SNP with physical activity in a random effects meta-analysis of 19,268 children and adolescents. (PDF) [file pmed.1001116.s009.pdf]
