# Supplementary material for: Physical Activity Attenuates the Influence of FTO Variants on Obesity Risk: A Meta-Analysis of 218,166 Adults and 19,268 Children
Source: PLoS Med. 2011 Nov 1;8(11):e1001116. doi: 10.1371/journal.pmed.1001116 (PMC3206047; doi:10.1371/journal.pmed.1001116)
Supplement: Figure S5 — Forest plot of the effect of the interaction between the FTO rs9939609 SNP and physical activity on body fat percentage in a random effects meta-analysis of 61,509 adults. (PDF) [file pmed.1001116.s005.pdf]

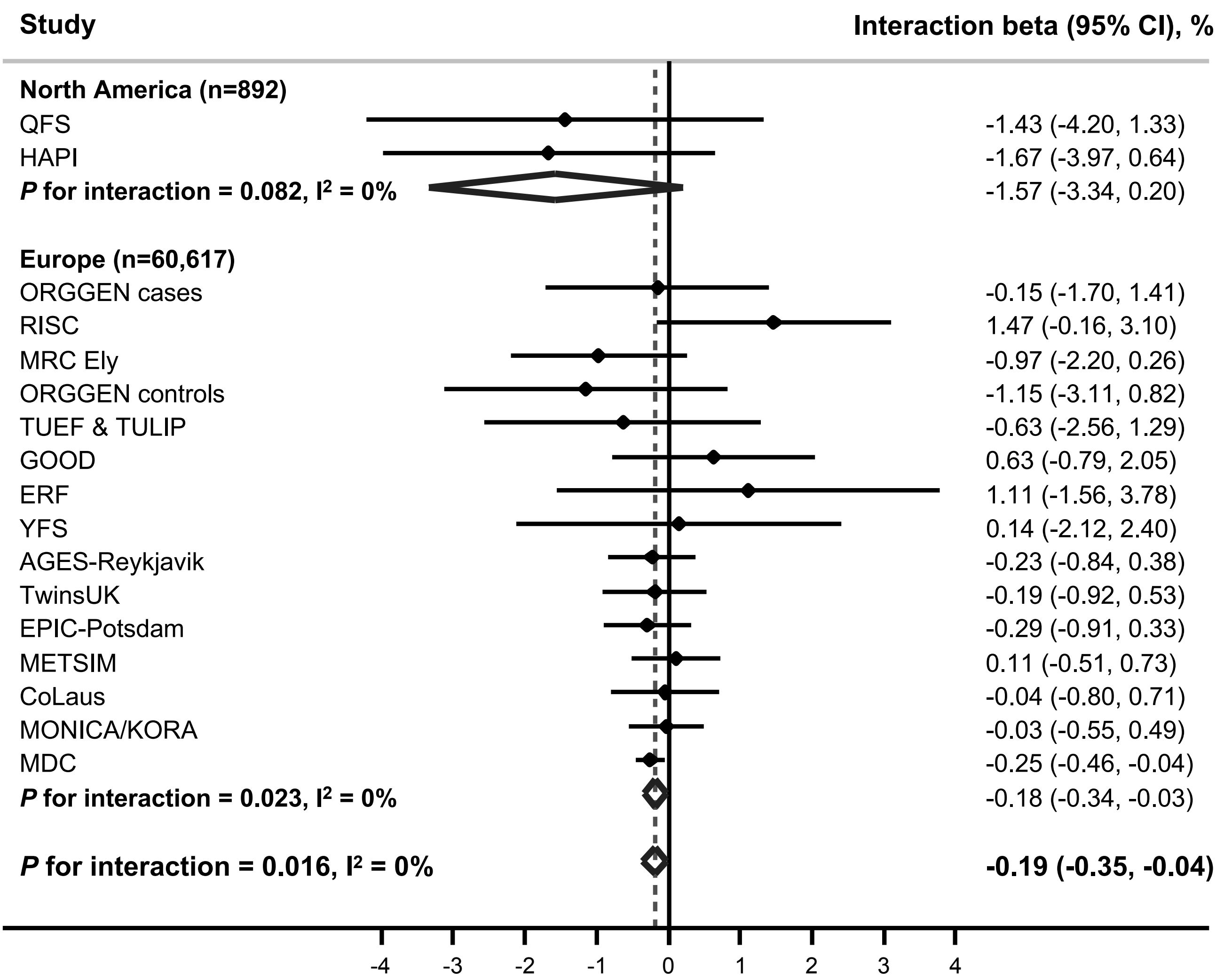
Study
Interaction beta (95% CI), %
North America (n=892)
QFS -1.43 (-4.20, 1.33)
HAPI -1.67 (-3.97, 0.64)
P for interaction = 0.082, I² = 0% -1.57 (-3.34, 0.20)
Europe (n=60,617)
ORGGEN cases -0.15 (-1.70, 1.41)
RISC 1.47 (-0.16, 3.10)
MRC Ely -0.97 (-2.20, 0.26)
ORGGEN controls -1.15 (-3.11, 0.82)
TUEF & TULIP -0.63 (-2.56, 1.29)
GOOD 0.63 (-0.79, 2.05)
ERF 1.11 (-1.56, 3.78)
YFS 0.14 (-2.12, 2.40)
AGES-Reykjavik -0.23 (-0.84, 0.38)
TwinsUK -0.19 (-0.92, 0.53)
EPIC-Potsdam -0.29 (-0.91, 0.33)
METSIM 0.11 (-0.51, 0.73)
CoLaus -0.04 (-0.80, 0.71)
MONICA/KORA -0.03 (-0.55, 0.49)
MDC -0.25 (-0.46, -0.04)
P for interaction = 0.023, I² = 0% -0.18 (-0.34, -0.03)
P for interaction = 0.016, I² = 0% -0.19 (-0.35, -0.04)
-4
-3
-2
-1
0
1
2
3
4
