# Supplementary material for: Physical Activity Attenuates the Influence of FTO Variants on Obesity Risk: A Meta-Analysis of 218,166 Adults and 19,268 Children
Source: PLoS Med. 2011 Nov 1;8(11):e1001116. doi: 10.1371/journal.pmed.1001116 (PMC3206047; doi:10.1371/journal.pmed.1001116)
Supplement: Table S1 — Association of physical activity with BMI, waist circumference, body fat percentage, risk of obesity, and risk of overweight in a random effects meta-analysis of up to 218,166 adults. (PDF) [file pmed.1001116.s010.pdf]

Table S1. Association of physical activity with BMI, waist circumference, body fat percentage, risk of obesity, and risk of overweight in a random effects meta-analysis of up to 218,166 adults.

Trait	Meta-analysis	N	beta (95% CI)	*P*	I^2
BMI (kg/m^2)	All individuals	218,166	-0.79 (-0.99, -0.60)	$2.7x10^{-15}$	94%
	Europe	164,307	-0.72 (-0.89, -0.55)	$4.3x10^{-16}$	91%
	North America	47,938	-1.34 (-1.83, -0.84)	$9.5x10^{-8}$	92%
	Asia	5,921	0.74 (0.06-1.43)	0.034	79%
Waist circumference (cm)	All individuals	159,848	-2.44 (-2.95, -1.93)	$1.1x10^{-20}$	92%
	Europe	128,811	-2.55 (-3.11, -1.98)	$1.1x10^{-18}$	92%
	North America	25,117	-3.19 (-4.34, -2.04)	$5.1x10^{-8}$	84%
	Asia	5,920	1.13 (-0.22, 2.49)	0.10	70%
Body fat percentage (%)	All individuals	61,509	-1.30 (-1.62, -0.98)	$1.1x10^{-15}$	77%
	Europe	60,617	-1.33 (-1.66, -1.00)	$2.1x10^{-15}$	80%
	North America	892	-0.70 (-1.98, 0.57)	0.279	0%
	Asia	NA	NA	NA	NA
		**N**	**OR (95% CI)**	***P***	I^2
Risk of obesity (BMI≥30 vs. BMI<25 kg/m^2)	All individuals	131,474	0.67 (0.60, 0.75)	$2.2x10^{-13}$	90%
	Europe	97,877	0.66 (0.58, 0.74)	$1.1x10^{-12}$	88%
	North America	29,282	0.58 (0.46, 0.72)	$1.3x10^{-6}$	86%
	Asia	4,315	1.66 (1.04, 2.66)	0.035	54%
Risk of overweight (BMI≥25 vs. BMI<25 kg/m^2)	All individuals	213,564	0.81 (0.76, 0.87)	$6.9x10^{-9}$	86%
	Europe	163,069	0.81 (0.76, 0.87)	$3.2x10^{-9}$	82%
	North America	44,574	0.70 (0.60, 0.83)	$1.6x10^{-5}$	82%
	Asia	5,921	1.23 (0.83, 1.81)	0.300	82%

All models are adjusted for age and sex. beta, difference in trait in physically active individuals compared to inactive individuals;

I^2, heterogeneity between studies in the association of physical activity with the trait; OR, odds ratio in physically active individuals compared with inactive individuals
