# Supplementary material for: Physical Activity Attenuates the Influence of FTO Variants on Obesity Risk: A Meta-Analysis of 218,166 Adults and 19,268 Children
Source: PLoS Med. 2011 Nov 1;8(11):e1001116. doi: 10.1371/journal.pmed.1001116 (PMC3206047; doi:10.1371/journal.pmed.1001116)
Supplement: Table S2 — Association of physical activity with age- and sex-standardized BMI, waist circumference, and body fat percentage in a random effects meta-analysis of up to 19,268 children and adolescents. (PDF) [file pmed.1001116.s011.pdf]

**Table S2.** Association of physical activity with age- and sex-standardised BMI, waist circumference, and body fat percentage in a random effects meta-analysis of up to 19,268 children and adolescents.

| **Trait** | **N** | **beta (95% CI)** | ***P*** | $I^2$ |
|---|---|---|---|---|
| BMI Z-score | 19,268 | -0.04 (-0.10, 0.02) | 0.15 | 39% |
| Waist circumference Z-score | 12,392 | -0.11 (-0.21, -0.00) | 0.044 | 60% |
| Body fat percentage Z-score | 6,864 | -0.21 (-0.38, -0.04) | 0.015 | 72% |

Beta, difference in trait in physically active children compared to inactive children; $I^2$, heterogeneity between studies in the association of physical activity with the trait
