# Supplementary material for: Physical Activity Attenuates the Influence of FTO Variants on Obesity Risk: A Meta-Analysis of 218,166 Adults and 19,268 Children
Source: PLoS Med. 2011 Nov 1;8(11):e1001116. doi: 10.1371/journal.pmed.1001116 (PMC3206047; doi:10.1371/journal.pmed.1001116)
Supplement: Table S3 — Association of the minor (A−) allele of the FTO rs9939609 SNP with age- and sex-standardized BMI, waist circumference, and body fat percentage in a random effects meta-analysis of up to 19,268 children and adolescents. (PDF) [file pmed.1001116.s012.pdf]

Table S3. Association of the minor (A) allele of the rs9939609 SNP in *FTO* with age- and sex-standardised BMI, waist circumference, and body fat percentage in a random effects meta-analysis of up to 19,268 children and adolescents.

	All children				Inactive children				Physically active children			
**Trait**	**N**	**beta (95% CI)**	***P***	**I^2**	**N**	**beta (95% CI)**	***P***	**I^2**	**N**	**beta (95% CI)**	***P***	**I^2**
BMI Z-score	19,268	0.10 (0.08, 0.12)	$1.3x10^{-21}$	0%	2,594	0.09 (0.03, 0.15)	0.0045	0%	16,674	0.10 (0.08, 0.12)	$1.3x10^{-18}$	0%
Waist circumference Z-score	8,638	0.11 (0.08, 0.13)	$8.0x10^{-16}$	0%	1,067	0.12 (0.03, 0.20)	0.0070	0%	7,571	0.10 (0.08, 0.13)	$9.6x10^{-13}$	0%
Body fat percentage Z-score	3,158	0.12 (0.09, 0.16)	$1.8x10^{-11}$	0%	306	0.17 (0.05, 0.30)	0.0059	0%	2,852	0.12 (0.06, 0.15)	$6.6x10^{-10}$	0%

All models are adjusted for age and sex.

beta, difference in trait per minor allele of rs9939609 or a proxy (r^2>0.8); I^2, heterogeneity between studies in the association of rs9939609 with the trait
