# Supplementary material for: Physical Activity Attenuates the Influence of FTO Variants on Obesity Risk: A Meta-Analysis of 218,166 Adults and 19,268 Children
Source: PLoS Med. 2011 Nov 1;8(11):e1001116. doi: 10.1371/journal.pmed.1001116 (PMC3206047; doi:10.1371/journal.pmed.1001116)
Supplement: Table S4 — Results of meta-regression showing the associations of all study characteristics combined with the FTO×PA interaction effect on BMI in adults. (PDF) [file pmed.1001116.s013.pdf]

Table S4. Results of meta-regression showing the associations of all study characteristics combined with the *FTO*×PA interaction effect on BMI in adults.

**Study characteristic**	**Effect ***	***P***
Sample size	+	0.41
Proportion of inactive individuals, %	+	0.56
Mean BMI, kg/m^2	-	0.99
Gender<br>*Male (0) vs. female (1)*	+	0.44
Age group<br>*<60 yrs (0) vs. ≥60 yrs (1)*	-	0.93
Study design<br>*population- or family-based (0), vs. case-control (1)*	-	0.63
Ethnicity<br>*White (0) vs. African American (1)*	+	0.51
Ethnicity<br>*White (0) vs. Asian (1)*	-	0.87
Ethnicity<br>*White (0) vs. Hispanic (1)*	-	0.30
Geographic region<br>*Europe (0) vs. North America (1)*	+	0.001
Geographic region<br>*Europe (0) vs. Asia (1)*	+	0.75
Measurement of PA<br>*PA variable categorical (0) vs. continuous (1)*	+	0.77
Measurement of PA,<br>*Leisure-time PA only (0)* vs. *leisure-time and occupational PA (1)*	+	0.22
Measurement of PA,<br>*Questionnaire-based (0)* vs. *objective (1)*	+	0.77

The results are for a meta-regression model where all the listed covariates were entered into the model simultaneously. Ethnicity and geographic region were entered into the model as indicator ('dummy') variables.

* Effect (+) indicates that an increase in the covariate value resulted as a stronger interaction between rs9939609 and PA (i.e. physically active individuals had a stronger attenuation in the association of rs9939609 risk allele with BMI), whereas effect (-) indicates the opposite.
