# Supplementary material for: Physical Activity Attenuates the Influence of FTO Variants on Obesity Risk: A Meta-Analysis of 218,166 Adults and 19,268 Children
Source: PLoS Med. 2011 Nov 1;8(11):e1001116. doi: 10.1371/journal.pmed.1001116 (PMC3206047; doi:10.1371/journal.pmed.1001116)
Supplement: Table S5 — Results of meta-regressions for the association of each study characteristic separately with the FTO×PA interaction effect on BMI in adults. (PDF) [file pmed.1001116.s014.pdf]

Table S5. Results of meta-regressions for the association of each study characteristic separately with the *FTO*xPA interaction effect on BMI in adults.

**Study characteristic**	**Effect ***	***P***
Sample size	+	0.17
Proportion of inactive individuals, %	-	0.46
Mean BMI, kg/m^2	+	0.64
Gender<br>*Male (0) vs. female (1)*	+	0.32
Age group<br>*<60 yrs (0) vs. ≥60 yrs (1)*	-	0.46
Study design<br>*population- or family-based (0), vs. case-control (1)*	-	0.80
Ethnicity<br>*White (0) vs. African American (1)*	+	0.21
Ethnicity<br>*White (0) vs. Asian (1)*	+	0.58
Ethnicity<br>*White (0) vs. Hispanic (1)*	-	0.50
Geographic region<br>*Europe (0) vs. North America (1)*	+	$3.3x10^{-6}$
Geographic region<br>*Europe (0) vs. Asia (1)*	+	0.43
Measurement of PA<br>*PA variable categorical (0) vs. continuous (1)*	+	0.10
Measurement of PA,<br>*Leisure-time PA only (0)* vs. *leisure-time and occupational PA (1)*	+	0.87
Measurement of PA,<br>*Questionnaire-based (0)* vs. *objective (1)*	+	0.41

The results are for metaregressions where each covariate was studied separately without adjustment for the other covariates. Ethnicity and geographic region were entered into the model as indicator ('dummy') variables.

* Effect (+) indicates that an increase in the covariate value resulted as a stronger interaction between rs9939609 and PA (i.e. physically active individuals had a stronger attenuation in the association of rs9939609 risk allele with BMI), whereas effect (-) indicates the opposite.
