# Supplementary material for: Physical Activity Attenuates the Influence of FTO Variants on Obesity Risk: A Meta-Analysis of 218,166 Adults and 19,268 Children
Source: PLoS Med. 2011 Nov 1;8(11):e1001116. doi: 10.1371/journal.pmed.1001116 (PMC3206047; doi:10.1371/journal.pmed.1001116)
Supplement: Text S1 — Supplementary descriptive information about the studies included in the meta-analyses. (PDF) [file pmed.1001116.s015.pdf]

# Physical activity attenuates the influence of *FTO* variants on obesity; a meta-analysis of 218,166 adults and 19,268 children

Tuomas O Kilpeläinen, Lu Qi, Soren Brage, Stephen J Sharp, Emily Sonestedt, *et al.**

* A full list of author names appears in the main paper.

---

# TEXT S1

---

**Text S1 - Table 1** Number of individuals, study design, and participant characteristics for studies of adults participating in the meta-analyses.

**Text S1 - Table 2** Number of individuals, study design, and participant characteristics for studies of children and adolescents participating in the meta-analyses.

**Text S1 - Table 3** Methods used for measuring BMI, waist circumference, and body fat percentage in studies of adults participating in the meta-analyses.

**Text S1 - Table 4** Methods used for measuring BMI, waist circumference, and body fat percentage in studies of children and adolescents participating in the meta-analyses.

**Text S1 - Table 5** Methods used for measuring physical activity and definitions of inactivity for studies of adults participating in the meta-analyses.

**Text S1 - Table 6** Methods used for measuring physical activity and definitions of inactivity for studies of children and adolescents participating in the meta-analyses.

**Text S1 - Table 7** Study-specific descriptive statistics in studies of adults participating in the meta-analyses.

**Text S1 - Table 8** Study-specific descriptive statistics in studies of children and adolescents participating in the meta-analyses.

**Text S1 - Table 9** Genotyping methods and quality control for the *FTO* SNP in all studies participating in the meta-analyses.

**Text S1 - Table 1.** Number of individuals, study design, and participant characteristics for studies of adults participating in the meta-analyses

| Study<br>Short name | Full name | Study design | Primary outcome | Total sample size | Participants | Analysis* | Reference PubMed ID |
|---|---|---|---|---|---|---|---|
| AGES-Reykjavik | Age/Gene Environment Susceptibility Reykjavik Study | Population-based cohort of adults born between 1907-1935 | Disease and disability in old age | 3,197 | white (1,347 men, 1,850 women) | Locally | 17351290 |
| ARIC | Atherosclerosis Risk in Communities | Multi-center cohort of 45-64-year old adults | Atherosclerosis and variation in cardiovascular risk factors | 10,890 | white (5,128 men, 5,762 women) | Locally | 2646917 |
| Birth Cohort 1958 | British Birth Cohort 1958 | Birth cohort | Health, educational and social development during lifecourse | 5,486 | white (2,614 men, 2,872 women) | Locally | 16155052 |
| BLSA | Baltimore Longitudinal Study of Aging | Population-based cohort of adults | Changes occurring with normal aging | 708 | white (326 men, 382 women) | Locally | Shock NW, et al. NIH publication no.84-2450 (1984). |
| BWHHS | British Women's Heart & Health Study | Cohort of 60-79-year-old women | Cardiovascular risk factors and incidence of cardiovascular disease | 3,107 | white women | Centrally | 12540690 |
| CLHNS | Cebu Longitudinal Health and Nutrition Survey | Cohort of women who gave birth in 1983-1984 | Maternal health | 1,711 | Filippino women | Locally | 20507864 |
| CoLaus | Cohorte Lausanneoise | Population-based, cross-sectional study of adults | Cardiovascular risk factors | 5,342 | white (2,530 men, 2,812 women) | Locally | 18366642 |
| DESIR | Data from an Epidemiological Study on the Insulin Resistance Syndrome | Cohort of middle-aged adults | Cardiovascular risk factors | 4,640 | white (2,308 men, 2,332 women) (~0.30% of overall DESIR population estimated to be of non-European ancestry; individuals born outside France excluded for the present study) | Locally | 9416436 |
| DPP | The Diabetes Prevention Program | Randomized controlled trial in overweight or obese adults | Incidence of type 2 diabetes | 3,364 | white (683 men, 1,267 women), African American (179 men, 510 women), Asian (83 men, 66 women), and Hispanic (193 men, 383 women) American-Indian individuals were excluded due to their small number. | Locally | 11832527 |

| Study Short name | Full name | Study design | Primary outcome | Total sample size | Participants | Analysis* | Reference PubMed ID |
|---|---|---|---|---|---|---|---|
| DPS | The Diabetes Prevention Study | Randomized controlled trial in overweight or obese adults | Incidence of type 2 diabetes | 487 | white (161 men, 326 women) | Centrally | 14633807 |
| ELSA | English Longitudinal Study of Ageing | Cohort of ≥50-year-old adults in the household sector in England | Health, economic position, and quality of life with ageing | 5,216 | white (2,394 men, 2,822 women) | Locally | |
| EPIC-NL | The European Prospective Investigation into Cancer and Nutrition - Netherlands Study | Population-based cohort of adults | Incidence of chronic disease | 1,729 | white (477 men, 1,252 women) | Locally | 19483199 |
| EPIC-Norfolk | The European Prospective Investigation into Cancer and Nutrition - Norfolk Study | Population-based cohort of adults | Incidence of chronic disease | 20,374 | white (10,059 men, 10,315 women) | Centrally | 10466767 |
| EPIC-Potsdam | The European Prospective Investigation into Cancer and Nutrition - Potsdam Study | Population-based cohort of adults | Incidence of chronic disease | 4,858 | white (1,917 men, 2,941 women) | Locally | 10592369 |
| ERF | Erasmus Rucphen Family Study | Family-based, cross-sectional study of a genetically isolated Dutch population | Risk of complex disease | 1,489 | white (655 men, 834 women) | Locally | 15054401 |
| FamHS | Family Heart Study | Multi-center cohort of individuals and families | Cardiovascular risk factors, preclinical atherosclerosis, and incidence of coronary heart disease | 839 | white (403 men, 436 women) | Locally | 19557197 |
| FUSION Stage 1 | Finland-United States Investigation of NIDDM Genetics Study Stage 1 | Case-control | Type 2 diabetes cases vs. controls with normal glucose tolerance | 1,955 | white (1,054 men, 901 women) | Locally | 17463248 |
| FUSION Stage 2 | Finland-United States Investigation of NIDDM Genetics Study Stage 2 | Case-control | Type 2 diabetes cases vs. controls with normal glucose tolerance | 977 | white (644 men, 333 women) | Locally | 17463248 |
| GLACIER | Gene-Lifestyle Interactions and Complex Traits Involved in Elevated Disease Risk | Population-based cohort of adults | Chronic disease incidence and risk factors | 14,828 | white (5,806 men, 9,022 women) | Locally | 20870969 |
| GOOD | The Gothenburg Osteoporosis and Obesity Determinants Study | Cohort of 18-20-year-old men | Bone and fat mass | 938 | white men | Centrally | 16007330 |
| HAPI | Heredity and Phenotype Intervention Heart Study | Controlled trial in a healthy, genetically isolated population of adults | Cardiovascular response to lifestyle intervention | 768 | Amish (408 men, 360 women) | Centrally | 18440328 |

| Study<br>Short name | Full name | Study design | Primary outcome | Total sample size | Participants | Analysis* | Reference PubMed ID |
|---|---|---|---|---|---|---|---|
| HUNT2-DiaB | The HUNT2-Bergen Diabetes Research Collaboration | Case-control | Type 2 diabetes cases vs. non-diabetic controls | 2,811 | white (1,455 men, 1,356 women) | Locally | 17827402 |
| InCHIANTI | InCHIANTI Study | Population-based cohort of adults | Physical functioning in late life | 1,132 | white (509 men, 623 women) | Locally | 11129752 |
| Inter99 | Inter99 Population-Based Cohort of Middle-Aged Danes | Randomized controlled trial in a population-based cohort of adults | Incidence of ischemic heart disease | 5,922 | white (2,891 men, 3,031 women) | Centrally | 14663300 |
| METSIM | The Metabolic Syndrome in Men Study | Population-based, cross-sectional study of 45-70-year-old men | Risk of type 2 diabetes and cardiovasular disease | 5,144 | white men | Locally | 19223598 |
| MDC | Malmö Diet and Cancer Cohort | Population-based cohort of adults | Incidence of cancer | 26,013 | white (10,200 men, 15,813 women) | Locally | 8429286<br>12028859 |
| MONICA/KORA | MONICA/KORA S2, S3 and S4 Surveys | Population-based cohort of adults | Cardiovascular risk factors | 11,679 | white (5,860 men, 5,819 women) | Locally | 16032512<br>16032514 |
| MPP | Malmö Preventive Project | Population-based cohort of adults | Total death and death from ischemic heart disease | 15,925 | white (10,330 men, 5,595 women) | Locally | 19373445 |
| MRC Ely | The MRC Ely Study | Population-based cohort of adults | Incidence of type 2 diabetes | 810 | white (336 men, 474 women) | Centrally | 17257284 |
| NFBC1966 | Northern Finland Birth Cohort 1966 | Birth cohort | Pre-term birth, low birth weight, and subsequent morbidity and mortality | 4,375 | white (2,106 men, 2,269 women) | Locally | 4911003 |
| NHS & HPFS | Nurses Health Study and Health Professionals Follow-up Study | Nested case-control | Type 2 diabetes cases vs. healthy controls | 5,453 | white (2,224 men, 3,229 women) | Locally | 18647953 |
| ORGGEN | Obesity Research Group - Genetics | Case-cohort | Obese cases vs. random controls from a population-based cohort | 1,629 | white men | Locally | 2508915<br>2361809 |
| PPP-Botnia | Prevalence, Prediction, and Prevention of Diabetes in Botnia Study | Population-based cohort of adults | Type 2 diabetes risk factors and prevalence | 2,291 | white (1,245 men, 1,046 women) | Locally | 19373445 |
| QFS | Quebec Family Study | Cohort of healthy parents and offspring | Fitness and risk factors for cardiovascular disease and diabetes | 711 | white (314 men, 397 women) | Centrally | 16355479 |

| Study Short name | Full name | Study design | Primary outcome | Total sample size | Participants | Analysis* | Reference PubMed ID |
|---|---|---|---|---|---|---|---|
| RISC | Relationship between Insulin Sensitivity and Cardiovascular Disease | Cohort of healthy adults | Insulin resistance and risk of cardiovascular disease | 771 | white (335 men, 436 women) | Centrally | 14968294 |
| Rotterdam | Rotterdam Study | Population-based cohort of adults ≥54 years of age | Diseases frequent in the elderly | 3,479 | white (1494 men, 1985 women) | Centrally | 19728115 1833235 |
| Segovia | The Segovia Study | Population-based, cross-sectional study of adults | Prevalence of anthropometric and physiological parameters related to metabolic syndrome | 586 | white (271 men, 315 women) | Centrally | Martínez-Larrad MT, et al. Med Clin (Barc). 2005;125:481-6. |
| Singapore NHS98 | 1998 Singapore National Health Survey | Population-based, cross-sectional study of adults | Prevalence of diabetes mellitus, hypertension, obesity, smoking, physical inactivity, and hypercholesterolaemia | 4,210 | Chinese (1,305 men, 1,559 women), Malay (369 men, 394 women), and Indian-Asian (282 men, 301 women) | Centrally | 18599522 |
| TUEF & TULIP | Tuebingen Family Study & Tuebingen Lifestyle Intervention Programme | TUEF: Controlled trial in healthy adults; TULIP: Controlled trial in adults with prediabetes phenotypes | TUEF: Incidence of type 2 diabetes and changes in adiposity; TULIP: Incidence of type 2 diabetes and improvement in prediabetes phenotypes | 930 | white (323 men, 607 women) | Centrally | 16205883 17327385 |
| TwinsUK | TwinsUK | Cohort of adult twin pairs | Cardiovascular, metabolic, musculoskeletal, dermatological, and opthalmological diseases | 3,964 | white (322 men, 3,642 women) | Locally | 12537873 19359265 |
| WGHS | Women's Genome Health Study | Cohort of US female health professionals | Incidence of chronic disease | 21,674 | white women | Locally | 18070814 |
| WHI-OS | The Women's Health Initiative - Observational Study | Nested case-control | Incident type 2 diabetes cases vs. healthy controls | 3,531 | white (n=1,818), African American (n=1,081), Asian (n=239), and Hispanic (n=393) women | Locally | 18787525 |
| YFS | The Cardiovascular Risk in Young Finns Study | Population-based cohort of adults | Cardiovascular risk from childhood to adulthood | 2,223 | white (997 men, 1226 women) | Locally | 18263651 |

*The analyses according to a standardized plan were either performed by each study 'locally' or the dataset was sent to the meta-analysts to be analysed 'centrally'.

**Text S1 - Table 2.** Number of individuals, study design, and participant characteristics for studies of children and adolescents participating in the meta-analyses

| Study<br>Short name | Full name | Study design | Primary outcome | Total sample size | Participants | Analysis* | Reference PubMed ID |
|---|---|---|---|---|---|---|---|
| ALSPAC | Avon Longitudinal Study of Parents and Children | Cohort of children | Child health and development | 3,760 | 10-13-year old whites (1814 boys 1,946 girls) | Locally | 11237119 |
| EYHS | European Youth Heart Study | Cross-sectional, multi-centre study of children and adolescents | Cardiovascular risk factors | 1,235 | 8-17-year old whites (550 boys, 685 girls) | Centrally | 15321796 |
| GENDAI | Gene-Diet Attica Investigation on Childhood Obesity | Cross-sectional, multi-centre study of children | Body weight | 913 | 9-13-year old whites (429 boys, 484 girls) | Locally | 17378724 |
| HELENA | Healthy Lifestyle in Europe by Nutrition in Adolescence | Cross-sectional, multi-centre study of adolescents | Nutrition and health in European adolescents | 755 | 11-18-year old whites (342 boys, 413 girls) | Locally | 20368485 |
| NFBC1966 | Northern Finland Birth Cohort 1966 | Birth cohort | Pre-term birth, low birth weight, and subsequent morbidity and mortality | 4,027 | 14-year old whites (1,877 boys, 2,150 girls) | Locally | 8494831 |
| NFBC1986 | Northern Finland Birth Cohort 1986 | Birth cohort | Maternal and child health | 5,468 | 16-year old whites (2,692 boys and 2,776 girls) | Locally | 4911003 |
| PANIC | The Physical Activity and Nutrition in Children Study | Controlled exercise and diet intervention trial in 6-8-year old children | Influence of lifestyle intervention on overweight and obesity | 472 | 6-8-year old whites (244 boys, 228 girls) | Centrally | 21054767 |
| STRIP | The Special Turku Coronary Risk Factor Intervention Project for Children | Controlled trial in healthy children | Influence of dietary counselling on dietary intakes, serum lipid and lipoprotein concentrations, and growth and development of infants and children | 382 | 14-15-year old whites (195 boys and 187 girls) | Locally | 18430753<br>19158205 |
| YFS | The Cardiovascular Risk in Young Finns Study | Population-based cohort of children and adolescents | Cardiovascular risk from childhood to adulthood | 2,256 | 3-18-year old whites (1008 boys and 1,248 girls) | Locally | 18263651 |

*The analyses according to a standardized plan were either performed by each study 'locally' or the dataset was sent to the meta-analysts to be analysed 'centrally'.

**Text S1 - Table 3.** Methods used for measuring BMI, waist circumference and body fat percentage in studies of adults participating in the meta-analyses

| Study | BMI | | Waist circumference | | Body fat percentage | | |
|---|---|---|---|---|---|---|---|
| | **Measured/ self-reported?** | **Measured on the same year with physical activity?** | **Measured/self-reported?** | **Measured on the same year with physical activity?** | **Measured/self-reported?** | **Measured on the same year with physical activity?** | **Method (instrument)** |
| AGES-Reykjavik | Measured | YES | Measured | YES | Measured | YES | Bioimpedance (Xitron HYDRA ECF/ICF, Model 4200) |
| ARIC | Measured | YES | Measured | YES | NA | NA | NA |
| Birth Cohort 1958 | Self-reported | YES | Measured | 3 years later | NA | NA | NA |
| BLSA | Measured | YES | Measured | YES | Measured | On different visit (results excluded from meta-analysis) | Skinfold thickness |
| BWHHS | Measured | YES | Measured | YES | NA | NA | NA |
| CLHNS | Measured | YES | Measured | YES | NA | NA | NA |
| CoLaus | Measured | YES | Measured | YES | Measured | YES | Bioimpedance (Bodystat 1500 Analyzer) |
| DESIR | Measured | YES | Measured | YES | Measured | 6 years later (results excluded from meta-analysis) | Bioimpedance (Tanita TBF-300P) |
| DPP | Measured | YES | Measured | YES | NA | NA | NA |
| DPS | Measured | YES | Measured | YES | NA | NA | NA |
| ELSA | Measured | YES | Measured | YES | NA | NA | NA |
| EPIC-NL | Measured | YES | Measured | YES | NA | NA | NA |
| EPIC-Norfolk | Measured | YES | Measured | YES | NA | NA | NA |
| EPIC-Potsdam | Measured | YES | Measured | YES | Measured | YES | Skinfold thickness |
| ERF | Measured | YES | Measured | YES | Measured | YES | DEXA (DPX Prodigy total body fan-beam densitometer) |
| FamHS | Measured | YES | Measured | YES | Measured | 6-9 years later (results excluded from meta-analysis) | Bioimpedance (RJL bioelectric impedance meter) |
| FUSION Stage 1 | Measured | YES | Measured | YES | NA | NA | NA |

| Study | BMI | | Waist circumference | | Body fat percentage | | |
|---|---|---|---|---|---|---|---|
| | Measured/ self-reported? | Measured on the same year with physical activity? | Measured/self-reported? | Measured on the same year with physical activity? | Measured/self-reported? | Measured on the same year with physical activity? | Method (instrument) |
| FUSION Stage 2 | Measured | YES | Measured | YES | NA | NA | NA |
| GLACIER | Measured | YES | NA | NA | NA | NA | NA |
| GOOD | Measured | YES | Measured | YES | Measured | YES | DEXA (Lunar Prodigy, GE Healthcare) |
| HAPI | Measured | YES | Measured | YES | Measured | On different visit (individuals with ≥3 years gap between measurements excluded) | DEXA (Hologic QDR-4500W) |
| HUNT2-DiaB | Measured | YES | Measured | YES | NA | NA | NA |
| InCHIANTI | Measured | YES | Measured | YES | NA | NA | NA |
| Inter99 | Measured | YES | Measured | YES | NA | NA | NA |
| MDC | Measured | YES | Measured | YES | Measured | YES | Bioimpedance (BIA 103 single-frequency analyzer) |
| METSIM | Measured | YES | Measured | YES | Measured | YES | Bioimpedance (Akern Bioimpedance Analyzer model BIA101) |
| MONICA/KORA | Measured | YES | Measured | YES | Measured | YES | Bioimpedance (KORA Survey 3: Body Composition Analyzer TVI-10; KORA Survey 4 and parts of Survey 3: B.I.A. 2000 Analyzer) |
| MPP | Measured | YES | NA | NA | NA | NA | NA |
| MRC Ely | Measured | YES | Measured | YES | Measured | YES | DEXA (GE Lunar Prodigy) |
| NFBC1966 | Measured | YES | Measured | YES | NA | NA | NA |
| NHS & HPFS | Self-reported | YES | Self-reported | YES | NA | NA | NA |
| ORGGEN | Measured | YES | Measured | YES | Measured | YES | Bioimpedance (BIA-103 RJL-system-analyzer) |
| PPP-Botnia | Measured | YES | NA | NA | NA | NA | NA |
| QFS | Measured | YES | Measured | YES | Measured | YES | Skinfold thickness |

| Study | BMI | | Waist circumference | | Body fat percentage | | |
|---|---|---|---|---|---|---|---|
| | Measured/ self-reported? | Measured on the same year with physical activity? | Measured/self-reported? | Measured on the same year with physical activity? | Measured/self-reported? | Measured on the same year with physical activity? | Method (instrument) |
| RISC | Measured | YES | Measured | YES | Measured | YES | Bioimpedance (Tanita TBF-300 Body Composition Analyzer) |
| Rotterdam | Measured | YES | Measured | YES | Measured | ~5 years later (results excluded from meta-analysis) | DEXA (Lunar Prodigy, GE Healthcare) |
| Segovia | Measured | YES | Measured | YES | NA | NA | NA |
| Singapore NHS98 | Measured | YES | Measured | YES | NA | NA | NA |
| TUEF & TULIP | Measured | YES | Measured | YES | Measured | YES | Bioimpedance (RJL Bioelectrical Impedance Analyzer) |
| TwinsUK | Measured | On different visit (individuals with ≥3 years gap between measurements excluded) | Measured | On different visit (individuals with ≥3 years gap between measurements excluded) | Measured | On different visit (individuals with ≥3 years gap between measurements excluded) | DEXA (Hologic Discover W' -QDR software ver. 12.6) |
| WGHS | Self-reported | YES | Self-reported | 6 years later (results excluded from meta-analysis) | NA | NA | NA |
| WHI-OS | Measured | YES | Measured | YES | NA | NA | NA |
| YFS | Measured | YES | Measured | YES | Measured | YES | Skinfold thickness |

* NA, data not available

**Text S1 - Table 4.** Methods used for measuring BMI, waist circumference and body fat percentage in studies of children and adolescents participating in the meta-analyses

| Study | BMI | | Waist circumference | | Body fat percentage | | |
|---|---|---|---|---|---|---|---|
| | **Measured/ self-reported?** | **Measured on the same year with physical activity?** | **Measured/self-reported?** | **Measured on the same year with physical activity?** | **Measured/self-reported?** | **Measured on the same year with physical activity?** | **Method (instrument)** |
| ALSPAC | Measured | YES | Measured | YES | Measured | YES | DEXA (Lunar Prodigy, GE Healthcare) |
| EYHS | Measured | YES | Measured | YES | Measured | YES | Skinfold thickness |
| GENDAI | Measured | YES | Measured | YES | Measured | YES | Skinfold thickness |
| HELENA | Measured | YES | Measured | YES | Measured | YES | Skinfold thickness |
| NFBC1966 | Self-reported | YES | NA | NA | NA | NA | NA |
| NFBC1986 | Measured | YES | Measured | YES | NA | NA | NA |
| PANIC | Measured | YES | Measured | YES | Measured | YES | Bioimpedance (InBody 720, Biospace) |
| STRIP | Measured | YES | Measured | YES | NA | NA | NA |
| YFS | Measured | YES | NA | NA | NA | NA | NA |

* NA, data not available

**Text S1 - Table 5.** Methods used for measuring physical activity and definitions of inactivity for studies of adults participating in the meta-analyses

| Study | PA measurement | PA measure used to define physical inactivity | Definition of physical inactivity |
|---|---|---|---|
| AGES-Reykjavik | Interviewer-administered questionnaire | The following question was used:<br>In the past 12 months, how often did you participate in moderate or vigorous physical activities? | Never or rarely participation in moderate or vigorous physical activities |
| ARIC | Self-administered questionnaire<br>(Baecke Questionnaire of Habitual Physical Activity) | Sport activity PA score | Sex-specific 20% with lowest PA |
| Birth Cohort 1958 | Self-administered questionnaire | The following questions were used:<br>1) Do you regularly take part in any of these activities; that is, at least once a month, for most of the year? (11 different activities listed)<br>2) Work class: manual, non-manual, unemployed/other | Response 'No' to question 1 and response 'non-manual' to question 2. |
| BLSA | Self-administered questionnaire | MET-minutes of leisure-time PA per week | Sex-specific 20% with lowest PA |
| BWHHS | Self-administered questionnaire | Hours of moderate-to-vigorous PA per typical week | Less than 1 h/wk of moderate-to-vigorous PA |
| CLHNS | Self-administered questionnaire | A) MET-minutes calculated by summing all individual activities during 24 hours of a typical day; B) MET-minutes calculated after grouping together similar tasks (e.g. personal hygiene) during 24 hours of a typical day | Women with PA in the lowest 20% of population when measured with both A and B. Women with PA in the lowest 40% of A or B but with PA in the lowest 20% of the other |
| CoLaus | Self-administered questionnaire | The following questions were used:<br>1) How much time (in minutes) do you walk every day on average to get to work, shops? 2) During working hours does your occupation involve: a) most often sitting, b) often standing, but little or no carrying or lifting of heavy loads, c) most often walking around and carrying light loads, d) important physical load, carrying heavy loads. 3) How many times per week do you take part for at least 20 minutes in leisure physical activities? | All of the following responses required:<br>1) <20 minutes of walking/day<br>2) 'Most often sitting' or 'Often standing, but little or no carrying or lifting of heavy loads' in occupation<br>3) Less than once a week of participation in 20 minutes of leisure PA |
| DESIR | Self-administered questionnaire | Number of times person was involved in structured PA per week | 0-1 times/week of structured PA |
| DPP | Self-administered questionnaire<br>(Modified Activity Questionnaire) | MET-min of leisure time PA per week | Sex-specific 20% with lowest PA for each ethnicity separately |
| DPS | Self-administered questionnaire<br>(Kuopio Ischemic Heart Disease Risk Factor Study 12-Month Leisure-Time Physical Activity Questionnaire) | MET-min of leisure time PA per week | Sex-specific 20% with lowest PA |

| Study | PA measurement | PA measure used to define physical inactivity | Definition of physical inactivity |
|---|---|---|---|
| ELSA | Self-administered questionnaire | The following questions were used:<br>1) Do you take part in sports or activities that are vigorous?<br>2) Do you take part in sports or activities that are moderately energetic? | Hardly ever participation in vigorous sports or activities and less than once a month of moderately energetic sports or activities |
| EPIC-NL | Self-administered questionnaire<br>(The EPIC Core Questionnaire) | Cambridge Physical Activity Index<br>(four categories: inactive, moderately inactive, moderately active, active) | Inactive category |
| EPIC-Norfolk | Self-administered questionnaire<br>(The EPIC Core Questionnaire) | Cambridge Physical Activity Index<br>(four categories: inactive, moderately inactive, moderately active, active) | Inactive category |
| EPIC-Potsdam | Self-administered questionnaire<br>(The EPIC Core Questionnaire) | Cambridge Physical Activity Index<br>(four categories: inactive, moderately inactive, moderately active, active) | Inactive category |
| ERF | Self-administered questionnaire | The following questions were used:<br>1) How would you describe the physical intensity of your work (light, fairly heavy, heavy or very heavy)?<br>2) How often do you practice sports with sweating or getting short of breath?<br>3) How often do you practice sports with slight sweating or slight increase in heart rate or breathing? | All of the following responses required:<br>1) Light intensity of work<br>2) Never or sometimes sports with sweating or getting short of breath<br>3) Never or sometimes sports with slight sweating or slight increase in heart rate or breathing |
| FamHS | Self-administered questionnaire | MET-minutes of leisure-time PA per week | Sex-specific 20% with lowest PA |
| FUSION Stage 1 and 2 | Self-administered questionnaire | The following questions were used:<br>1) How strenuous is your work?<br>2) How much do you exercise or exert yourself in your spare time? | Both of the following responses required:<br>1) 'I have not worked because I am retired or not working for other reasons' or 'light deskwork'<br>2) 'In my spare time I read, watch TV, and work in the household tasks which don't make me move much or physically tax me' |
| GLACIER | Self-administed questionnaire<br>(Umeå EPIC Questionnaire) | Frequency of structured exercise (coded as exercise never/rarely vs. one or more times weekly) | Exercise never/rarely |
| GOOD | Self-administered questionnaire | Hours of recreational PA per week | No participation in recreational PA |
| HAPI | Accelerometer<br>(Actical ver. 8.2 or 8.3 worn seven full days) | Mean accelerometer counts per minute worn | Sex-specific 20% with lowest PA |

| Study | PA measurement | PA measure used to define physical inactivity | Definition of physical inactivity |
|---|---|---|---|
| HUNT2-DiaB | Self-administered questionnaire | The following questions were used:<br>1) Number of times per week with light activity (not sweaty/breathless)<br>2) Number of times per week with hard physical activity (sweaty/breathless)<br>3) How will you describe your physical activity at work? | All of the following responses required:<br>1) Below one time per week of light activity<br>2) No hard activity per week<br>3) Mostly sedentary work.<br>If there was no response on question 3, only questions 1 and 2 were used |
| InCHIANTI | Self-administered questionnaire | The following question was used:<br>What was your physical activity level last year (hardly any physical activity, mostly sitting/some walking, light exercise 2-4 h/wk, moderate 1-2 h/wk or light exercise >4 h/wk, or moderate exercise > 3 h/wk)? | Response 'Hardly any physical activity' or 'Mostly sitting/some walking' |
| Inter99 | Self-administered questionnaire | A combined PA score (three categories: physically passive, light or medium physically active, hard or very hard physically active) calculated based on responses to two questions; one enquiring about the level of physical activity at work and the other of leisure-time PA | Physically passive category |
| MDC | Self-administered questionnaire | MET-minutes of PA per week | Sex-specific 20% with lowest PA |
| METSIM | Self-administered questionnaire | The following questions were used:<br>1) Level of physical activity at work (light/moderate/heavy)<br>2) Level of leisure-time physical activity ('very low' = almost completely inactive, 'low' = some minor physical activity, 'moderate' = physical activity ≤2 times/wk ≥30 min, 'high' = physical activity ≥3 times/wk ≥30 min) | Both of the following responses required:<br>1) Light physical activity at work<br>2) 'Very low' or 'low' leisure-time PA |
| MONICA/KORA | Self-administered questionnaire | The following questions were used:<br>1) How often do you carry out sports in the winter?<br>2) How often do you carry out sports in the summer?<br>3) How would you classify your work or main employment? | Less than 1 h/wk of sports in the winter and summer and 'no appreciable physical work' in main employment |
| MPP | Self-administered questionnaire | The following questions were used:<br>1) Do you walk or cycle to and from work?<br>2) Do you walk or cycle for recreation during weekdays?<br>3) Do you walk or cycle for recreation during weekend days?<br>4) Do you undertake at least 3 h/wk of structured physical exercise?<br>5) Do you walk to work or do yard work?<br>6) Do you perform light structured physical exercise each week? | Response 'No' to all questions |
| MRC Ely | Heart rate sensor<br>(Flex Heart Rate technique) | Physical Activity Level (total daily energy expenditure divided by resting energy expenditure) | Sex-specific 20% with lowest PA |
| NFBC1966 | Self-administered questionnaire | MET-hours of brisk PA per week | Sex-specific 20% with lowest PA |

| Study | PA measurement | PA measure used to define physical inactivity | Definition of physical inactivity |
|---|---|---|---|
| NHS & HPFS | Self-administered questionnaire | MET-hours of leisure-time PA per week | Sex-specific 20% with lowest PA |
| ORGGEN | Self-administered questionnaire | Level of PA defined by the following categories:<br>1) Being almost entirely inactive or engaging in light PA less than 2 hours/week<br>2) Engaging in light PA 2-4 hours/week<br>3) Engaging in light PA more than 4 hours/week or in vigorous PA 2-4 hours/week<br>4) engaging in highly vigorous PA more than 4 hours/week in regular heavy exercise or competitive sports several times per week | Being almost entirely inactive or engaging in light PA less than 2 hours/week |
| PPP-Botnia | Self-administered questionnaire<br>(Kuopio Ischemic Heart Disease Risk Factor Study 12-Month Leisure-Time Physical Activity Questionnaire) | MET-min of leisure time PA per week | Sex-specific 20% with lowest PA |
| QFS | Bouchard 3-day physical activity diary | Total daily activity level | Sex-specific 20% with lowest PA |
| RISC | Accelerometer<br>(Actigraph) | Mean accelerometer counts per minute worn | Sex-specific 20% with lowest PA |
| Rotterdam | Self-administered questionnaire | MET-minutes of leisure-time PA per week | Sex-specific 20% with lowest PA |
| Segovia | Self-administered questionnaire | MET-hours of regular leisure-time PA per week | Sex-specific 20% with lowest PA |
| Singapore NHS98 | Interviewer-administered questionnaire | The following questions were used:<br>1) Level of occupational physical activity (sedentary/light/moderate/heavy)<br>2) On average, how many times do you participate in sports, exercise or brisk walking in a week (each session should last for at least 20 minutes)? | Sedentary occupation and no participation in ≥20 min of sports, exercise or brisk walking |
| TUEF & TULIP | Self-administered questionnaire<br>(Baecke Questionnaire of Habitual Physical Activity) | Total habitual PA score | Sex-specific 20% with lowest PA |
| TwinsUK | Self-administered questionnaire | The following questions were used:<br>1) During the last 12 months, how would you describe the kind of physical activity at work? (inactive/light/moderate/heavy/not applicable)<br>2) In the past year, how frequently have you typically engaged in physical exercise that raise your heart rate and last for 20 minutes at a time? | Inactive' or 'light' PA at work and less than once a month of physical exercise that raised heart rate and lasted for 20 minutes at a time |

| Study | PA measurement | PA measure used to define physical inactivity | Definition of physical inactivity |
|---|---|---|---|
| WGHS | Self-administered questionnaire | MET-hours of leisure-time PA per week | Sex-specific 20% with lowest PA for each ethnicity separately |
| WHI-OS | Self-administered questionnaire | MET-hours of recreational PA per week | Sex-specific 20% with lowest PA |
| YFS | Self-administered questionnaire | The following question was used:<br>How often do you engage in rigorous physical activity? | Less than once a week |

MET, metabolic equivalent; PA, physical activity

**Text S1 - Table 6.** Methods used for measuring physical activity and definitions of inactivity for studies of children and adolescents participating in the meta-analyses

| Study | PA measurement | PA measure used to define physical inactivity | Definition of physical inactivity |
|---|---|---|---|
| ALSPAC | Accelerometer<br>(Actigraph) | Mean accelerometer counts per minute worn | Sex- and age-specific 10% with lowest PA |
| EYHS | Accelerometer<br>(MTI model WAM 7164 worn 2 weekdays and 2 weekend days) | Mean accelerometer counts per minute worn | Sex- and age-specific 10% with lowest PA |
| GENDAI | Self-administered PA recall checklist | MET-minutes of PA per day | Sex- and age-specific 10% with lowest PA |
| HELENA | Accelerometer<br>(Actigraph worn seven full consecutive days) | Mean accelerometer counts per minute worn | Sex- and age-specific 10% with lowest PA |
| NFBC1966 | Self-administered questionnaire | The following question was used:<br>How often do you participate in sports after school hours? | Response 'once in two weeks', 'once a month' or 'usually never' |
| NFBC1986 | Self-administered questionnaire | The following question was used:<br>Outside school hours, how many hours a week do you spend on brisk physical activity? | Less than 1h/wk of brisk PA |
| PANIC | Self-administered questionnaire<br>(completed by the parents on behalf of their child) | The following measures were used:<br>1) Distance from home to school commuted by foot or by bicycle.<br>2) Number of exercise sessions/wk outside school hours. | Commuting to school <3 km/week by foot or <5 km/week by bicycle, and 0-2 exercise sessions/wk outside school hours |
| STRIP | Self-administered questionnaire | MET-hours of leisure-time PA per week | Sex- and age-specific 10% with lowest PA |
| YFS | Self-administered questionnaire | The following question was used:<br>How often do you engage in leisure-time physical activity for at least half an hour per session? | Less than once a week of leisure-time PA ≥30 min |

MET, metabolic equivalent; PA, physical activity

**Text S1 - Table 7.** Study-specific descriptive statistics in studies of adults participating in the meta-analyses

| Study | Men | | | | | | Women | | | | | |
|---|---|---|---|---|---|---|---|---|---|---|---|---|
| | N | Inactive (%) | Age, yrs mean (sd) | BMI, kg/m² mean (sd) | Waist circumference, cm mean (sd) | Fat% mean (sd) | N | Inactive (%) | Age, yrs mean (sd) | BMI, kg/m² mean (sd) | Waist circumference, cm mean (sd) | Fat% mean (sd) |
| AGES-Reykjavik | 1,347 | 59.0 | 76.7 (5.3) | 27.0 (3.8) | 102.5 (10.5) | 22.0 (5.5) | 1,850 | 66.3 | 76.7 (5.8) | 27.2 (4.9) | 99.6 (13.2) | 34.0 (5.0) |
| ARIC | 5,128 | 19.5 | 54.8 (5.7) | 27.4 (4.0) | 99.7 (10.4) | - | 5,762 | 16.8 | 53.9 (5.7) | 26.6 (5.5) | 93.1 (14.9) | - |
| Birth Cohort 1958 | 2,614 | 10.6 | 42.0 (0.0) | 26.5 (3.9) | 98.5 (11.1) | - | 2,872 | 17.4 | 42.0 (0.0) | 24.8 (4.7) | 84.8 (12.5) | - |
| BLSA | 326 | 19.9 | 50.1 (17.3) | 23.9 (3.7) | 76.6 (9.3) | - | 382 | 19.9 | 48.6 (16.8) | 25.3 (3.1) | 89.4 (9.2) | - |
| BWHHS | 0 | - | - | - | - | - | 3,107 | 52.6 | 68.9 (5.5) | 27.5 (5.0) | 86.0 (12.2) | - |
| CLHNS | 0 | - | - | - | - | - | 1,711 | 21.5 | 48.4 (6.1) | 24.3 (4.4) | 81.1 (10.9) | - |
| CoLaus | 2,530 | 13.0 | 53.2 (10.9) | 26.6 (4.2) | 88.7 (13.6) | 23.9 (6.0) | 2,812 | 11.2 | 54.3 (10.7) | 25.1 (4.9) | 89.8 (13.2) | 34.4 (8.2) |
| DESIR | 2,308 | 49.4 | 47.2 (10.1) | 25.4 (3.3) | 89.7 (9.6) | - | 2,332 | 53.9 | 47.5 (9.9) | 24.0 (4.1) | 77.1 (10.4) | - |
| DPP African American | 179 | 19.6 | 55.1 (10.0) | 32.6 (5.9) | 106.9 (14.1) | - | 510 | 19.8 | 49.1 (9.4) | 36.2 (7.0) | 106.2 (15.3) | - |
| DPP Asian | 83 | 19.3 | 51.5 (10.0) | 28.5 (3.7) | 97.1 (9.4) | - | 66 | 19.7 | 47.7 (6.5) | 31.0 (6.7) | 93.5 (14.2) | - |
| DPP Hispanic | 193 | 19.7 | 51.8 (10.9) | 31.4 (4.9) | 104.6 (12.4) | - | 383 | 19.8 | 47.5 (9.8) | 34.0 (5.9) | 99.7 (12.5) | - |
| DPP white | 683 | 19.6 | 54.0 (10.5) | 32.5 (5.8) | 110.4 (13.3) | - | 1,267 | 20.0 | 50.9 (10.3) | 35.0 (7.0) | 104.3 (14.7) | - |
| DPS | 161 | 19.9 | 55.4 (7.6) | 29.9 (3.5) | 104.2 (9.8) | - | 326 | 19.9 | 55.1 (7.0) | 31.9 (4.8) | 99.8 (11.3) | - |
| ELSA | 2,394 | 54.9 | 63.3 (9.1) | 27.7 (4.1) | 101.0 (11.1) | - | 2,822 | 67.2 | 63.6 (9.4) | 27.8 (5.2) | 90.4 (12.2) | - |
| EPIC-NL | 477 | 10.7 | 44.5 (11.1) | 26.1 (3.7) | 93.1 (11.5) | - | 1,252 | 8.1 | 51.4 (11.6) | 26.2 (4.1) | 83.0 (10.6) | - |
| EPIC-Norfolk | 10,059 | 30.5 | 59.1 (9.3) | 26.5 (3.3) | 95.7 (9.7) | 23.5 (6.1) | 10,315 | 30.9 | 58.5 (9.3) | 26.1 (4.2) | 82.1 (10.7) | 39.6 (9.0) |
| EPIC-Potsdam | 1,917 | 17.2 | 51.7 (8.1) | 27.0 (3.6) | 94.7 (10.0) | 24.5 (5.5) | 2,941 | 17.7 | 48.5 (9.1) | 25.8 (4.8) | 80.8 (11.8) | 35.2 (5.9) |
| ERF | 655 | 4.1 | 49.1 (13.7) | 27.0 (4.0) | 93.2 (11.5) | 26.7 (7.1) | 834 | 3.6 | 47.8 (13.9) | 26.3 (4.6) | 81.1 (11.4) | 38.5 (7.5) |
| FamHS | 403 | 17.6 | 53.1 (12.1) | 28.1 (4.2) | 101.1 (11.0) | - | 436 | 22.9 | 57.7 (9.1) | 27.5 (5.8) | 96.0 (15.2) | - |
| FUSION Stage 1 cases | 605 | 16.7 | 62.7 (7.1) | 29.5 (4.0) | 104.3 (10.8) | - | 455 | 29.0 | 64.1 (7.9) | 31.2 (5.3) | 99.0 (12.8) | - |
| FUSION Stage 1 controls | 449 | 12.0 | 61.3 (7.5) | 27.0 (3.3) | 96.2 (9.7) | - | 446 | 10.1 | 61.5 (7.3) | 27.2 (4.2) | 86.2 (10.9) | - |
| FUSION Stage 2 cases | 366 | 21.6 | 56.4 (8.7) | 30.9 (5.1) | 105.9 (13.6) | - | 202 | 26.7 | 59.9 (7.2) | 32.2 (5.9) | 98.2 (13.8) | - |
| FUSION Stage 2 controls | 278 | 9.0 | 54.6 (6.4) | 27.0 (3.5) | 94.1 (9.8) | - | 131 | 13.0 | 60.5 (3.8) | 27.7 (4.2) | 83.2 (9.3) | - |
| GLACIER | 5,806 | 48.0 | 53.0 (9.7) | 26.3 (4.1) | - | - | 9,022 | 48.4 | 51.7 (8.2) | 25.7 (4.1) | - | - |
| GOOD | 938 | 36.1 | 18.9 (0.6) | 22.4 (3.2) | 78.5 (7.2) | 17.1 (7.4) | 0 | - | - | - | - | - |
| HAPI | 408 | 19.9 | 42.6 (13.6) | 25.6 (3.2) | 90.1 (9.9) | 19.3 (6.2) | 360 | 20.0 | 45.6 (14.0) | 27.8 (5.4) | 84.3 (11.3) | 35.4 (6.3) |
| HUNT2-DiaB cases | 461 | 16.7 | 61.3 (15.0) | 28.0 (3.9) | 97.3 (10.9) | - | 404 | 29.0 | 63.4 (15.7) | 30.0 (6.0) | 92.7 (13.6) | - |
| HUNT2-DiaB controls | 994 | 10.1 | 51.8 (17.4) | 26.5 (3.5) | 92.2 (9.2) | - | 952 | 15.1 | 50.7 (18.0) | 26.3 (4.5) | 81.6 (11.2) | - |
| InCHIANTI | 509 | 8.6 | 67.3 (14.9) | 27.0 (3.4) | 94.5 (9.4) | - | 623 | 19.9 | 68.4 (15.7) | 27.3 (4.7) | 88.8 (11.9) | - |

| | | | | | | | | | | | | |
|---|---|---|---|---|---|---|---|---|---|---|---|---|
| Inter99 | 2,891 | 30.8 | 46.3 (7.7) | 26.8 (4.0) | 93.2 (11.0) | - | 3,031 | 38.9 | 45.7 (7.8) | 25.8 (5.1) | 80.3 (11.9) | - |
| MDC | 10,200 | 20.0 | 59.1 (7.1) | 26.3 (3.5) | 93.8 (10.1) | 20.7 (5.0) | 15,813 | 20.0 | 57.3 (7.9) | 25.5 (4.2) | 77.9 (11.4) | 30.8 (5.0) |
| METSIM | 5,144 | 12.5 | 56.5 (6.7) | 26.8 (3.7) | 97.0 (10.5) | 23.0 (6.0) | 0 | - | - | - | - | - |
| MONICA/KORA | 5,860 | 15.3 | 49.7 (14.1) | 27.3 (3.8) | 96.1 (10.6) | 28.5 (6.3) | 5,819 | 8.8 | 49.0 (13.8) | 26.6 (5.1) | 83.4 (12.3) | 36.4 (6.9) |
| MPP | 10,330 | 22.0 | 42.9 (5.9) | 24.4 (2.6) | - | - | 5,595 | 21.1 | 50.2 (8.7) | 24.0 (4.6) | - | - |
| MRC Ely | 336 | 19.9 | 53.8 (11.1) | 26.7 (3.2) | 94.9 (9.7) | 23.9 (4.6) | 474 | 19.8 | 53.4 (10.3) | 26.2 (4.6) | 81.4 (10.9) | 37.0 (6.4) |
| NFBC1966 | 2,106 | 22.7 | 31.0 (0.0) | 25.2 (3.6) | 88.9 (9.9) | - | 2,629 | 20.2 | 31.0 (0.0) | 24.2 (4.8) | 79.1 (12.2) | - |
| NHS & HPFS cases | 1,040 | 24.0 | 55.6 (8.4) | 27.8 (4.1) | 101.7 (11.1) | - | 1,384 | 24.1 | 54.1 (6.8) | 29.8 (6.0) | 90.1 (13.7) | - |
| NHS & HPFS controls | 1,184 | 16.1 | 55.7 (8.5) | 25.1 (2.8) | 94.6 (8.4) | - | 1,845 | 16.9 | 53.8 (6.8) | 25.6 (5.0) | 79.1 (11.2) | - |
| ORGGEN cases | 751 | 16.2 | 43.6 (6.3) | 35.7 (5.7) | 117.2 (14.2) | 34.5 (5.2) | 0 | - | - | - | - | - |
| ORGGEN controls | 878 | 9.2 | 48.1 (8.4) | 26.1 (3.6) | 93.5 (10.5) | 24.5 (5.8) | 0 | - | - | - | - | - |
| PPP-Botnia | 1,245 | 19.9 | 45.4 (14.9) | 25.3 (4.4) | - | - | 1,046 | 20.0 | 46.4 (16.2) | 26.4 (4.0) | - | - |
| QFS | 314 | 19.7 | 40.4 (15.9) | 27.3 (6.4) | 93.5 (16.6) | 22.8 (9.0) | 397 | 18.9 | 40.3 (13.8) | 27.5 (8.1) | 83.8 (18.2) | 32.0 (10.1) |
| RISC | 335 | 19.7 | 44.2 (8.2) | 26.0 (3.1) | 92.7 (10.0) | 21.4 (6.2) | 436 | 20.0 | 45.7 (8.0) | 24.5 (4.2) | 80.9 (11.1) | 32.0 (7.6) |
| Rotterdam | 1,494 | 19.0 | 71.8 (6.3) | 26.3 (3.2) | 97.5 (9.4) | - | 1,985 | 18.9 | 73.0 (7.3) | 27.3 (4.4) | 90.7 (12.3) | - |
| Segovia | 271 | 16.2 | 54.7 (12.9) | 27.4 (3.4) | 95.7 (9.0) | - | 315 | 19.0 | 56.4 (11.8) | 27.5 (4.6) | 85.7 (11.1) | - |
| Singapore NHS98 Chinese | 1,305 | 12.3 | 38.3 (12.0) | 23.5 (3.7) | 84.1 (9.9) | - | 1,559 | 26.4 | 36.9 (12.0) | 22.0 (3.6) | 73.1 (8.5) | - |
| Singapore NHS98 Indian Asian | 282 | 14.5 | 40.6 (12.0) | 24.5 (4.1) | 88.8 (10.5) | - | 301 | 13.3 | 40.8 (12.9) | 25.6 (5.0) | 81.7 (11.6) | - |
| Singapore NHS98 Malay | 369 | 8.7 | 39.7 (11.7) | 24.9 (4.2) | 86.2 (10.7) | - | 394 | 17.3 | 38.1 (11.9) | 26.4 (5.5) | 79.5 (11.6) | - |
| TUEF & TULIP | 323 | 18.3 | 40.2 (13.6) | 26.8 (5.0) | 95.5 (14.0) | 21.9 (6.8) | 607 | 18.6 | 39.6 (12.6) | 27.0 (6.0) | 88.0 (14.3) | 32.9 (8.7) |
| TwinsUK | 322 | 25.8 | 50.5 (14.9) | 26.7 (3.9) | 93.2 (9.5) | 23.3 (5.6) | 3,642 | 29.8 | 50.0 (13.3) | 25.9 (4.9) | 79.5 (10.5) | 34.2 (7.0) |
| WGHS | 0 | - | - | - | - | - | 21,674 | 20.2 | 54.2 (7.1) | 25.9 (5.0) | - | - |
| WHI-OS African American | 0 | - | - | - | - | - | 1,081 | 21.3 | 61.2 (6.7) | 30.9 (7.1) | 91.0 (14.6) | - |
| WHI-OS Asian | 0 | - | - | - | - | - | 239 | 20.1 | 62.8 (8.1) | 24.7 (4.5) | 78.2 (11.0) | - |
| WHI-OS Hispanic | 0 | - | - | - | - | - | 393 | 22.9 | 60.2 (6.8) | 29.0 (5.8) | 87.3 (13.9) | - |
| WHI-OS white | 0 | - | - | - | - | - | 1,818 | 19.6 | 63.9 (6.8) | 29.5 (6.8) | 91.2 (16.1) | - |
| YFS | 997 | 29.1 | 31.7 (5.0) | 25.7 (4.1) | 89.8 (10.8) | 16.1 (6.0) | 1,226 | 22.2 | 31.5 (5.0) | 24.5 (4.6) | 79.4 (11.4) | 27.0 (7.6) |

**Text S1 - Table 8.** Study-specific descriptive statistics in children and adolescents participating in the meta-analyses

| Study | Boys | | | | | | Girls | | | | | |
|---|---|---|---|---|---|---|---|---|---|---|---|---|
| | N | Inactive (%) | Age, yrs mean (sd) | BMI, kg/m² mean (sd) | Waist circumference, cm mean (sd) | Fat% mean (sd) | N | Inactive (%) | Age, yrs mean (sd) | BMI, kg/m² mean (sd) | Waist circumference, cm mean (sd) | Fat% mean (sd) |
| ALSPAC | 1,814 | 9.5 | 11.7 (0.2) | 18.7 (3.1) | 68.3 (9.3) | 22.8 (9.4) | 1,946 | 9.9 | 11.7 (0.2) | 19.2 (3.4) | 67.7 (9.0) | 27.9 (8.5) |
| EYHS | 550 | 9.8 | 11.5 (2.7) | 18.2 (2.8) | 63.4 (8.0) | 16.3 (4.6) | 685 | 10.1 | 11.7 (2.8) | 18.3 (3.0) | 61.5 (7.4) | 20.9 (5.2) |
| GENDAI | 429 | 11.7 | 11.2 (0.7) | 20.3 (3.5) | 70.3 (9.7) | 25.9 (10.4) | 484 | 10.1 | 11.1 (0.6) | 19.8 (3.4) | 67.2 (9.2) | 31.3 (11.7) |
| HELENA | 342 | 9.9 | 14.5 (1.4) | 20.6 (3.3) | 72.7 (7.9) | 19.3 (10.6) | 413 | 9.9 | 14.4 (1.3) | 21.1 (3.4) | 70.6 (8.0) | 26.0 (7.2) |
| NFBC1966 | 1,877 | 17.2 | 14.0 (0.0) | 19.1 (2.4) | - | - | 2,150 | 28.9 | 14.0 (0.0) | 19.4 (2.7) | - | - |
| NFBC1986 | 2,692 | 11.9 | 16.0 (0.0) | 21.5 (4.0) | 76.0 (9.2) | - | 2,776 | 12.2 | 16.0 (0.0) | 21.0 (3.6) | 72.1 (8.1) | - |
| PANIC | 244 | 7.0 | 7.6 (0.4) | 16.1 (2.0) | 57.1 (5.2) | 14.6 (6.3) | 228 | 10.1 | 7.6 (0.4) | 16.1 (2.2) | 56.0 (5.8) | 18.2 (7.0) |
| STRIP | 195 | 9.7 | 15.0 (0.0) | 20.3 (3.2) | 74.6 (8.8) | - | 187 | 10.2 | 15.0 (0.1) | 20.6 (3.1) | 70.9 (7.5) | - |
| YFS | 1,008 | 9.0 | 10.4 (5.0) | 19.1 (3.2) | - | - | 1,248 | 9.5 | 10.5 (5.0) | 19.4 (3.2) | - | - |

**Text S1 - Table 9.** Genotyping methods and quality control for the *FTO* SNP in all studies participating in the meta-analyses

| Cohort | SNP | $r^2$ with rs9939609* | Minor allele | Genotyping Platform | Call rate* | Duplicate concordance | *p* for HWE |
|---|---|---|---|---|---|---|---|
| AGES-Reykjavik | rs3751812 | 1 | T | Illumina 370CNV BeadChip | 97% | NA | 0.57 |
| ALSPAC | rs9939609 | 1 | A | Genotyped by Kbioscience (http://www.kbioscience.co.uk) | NA | NA | 0.49 |
| ARIC | rs9939609 | - | A | TaqMan® SNP Genotyping Assays (Applied Biosystems) | 95% | 97% | 0.68 |
| Birth Cohort 1958 | rs9939609 | - | A | TaqMan® SNP Genotyping Assays (Applied Biosystems) | 98% | NA | 0.94 |
| BLSA | rs8050136 | 1 | A | Illumina 550K chip | 99% | NA | 0.47 |
| BWHHS | rs9939609 | - | A | Genotyped by Kbioscience (http://www.kbioscience.co.uk) | 95% | 99% | 0.91 |
| CLHNS | rs9939609 | - | A | TaqMan® SNP Genotyping Assays (Applied Biosystems) | 97% | 100% | 0.27 |
| CoLaus | rs9939609 | - | A | Affymetrix 500K chip | 99% | NA | 0.75 |
| DESIR | rs1421085 | 0.93 | C | TaqMan® SNP Genotyping Assays (Applied Biosystems) | 97% | 100% | 0.81 |
| DPP | rs9939609 | - | A | iPLEX™ Sequenom MassARRAY® | 97% | NA | White: 0.70<br>African American: 0.99<br>Asian: 0.20<br>Hispanic: 0.83 |
| DPS | rs9939609 | - | A | Illumina Golden Gate Assay | 99% | NA | 0.78 |
| ELSA | rs9939609 | - | A | TaqMan® SNP Genotyping Assays (Applied Biosystems) | 99% | 100% | 0.68 |
| EPIC-NL | rs9939609 | - | A | Illumina IBC v.3. chip | 98% | NA | 0.91 |
| EPIC-Norfolk | rs1121980 | 0.84 | T | TaqMan® SNP Genotyping Assays (Applied Biosystems) | 98% | 99% | 0.90 |
| EPIC-Potsdam | rs9935401 | 1 | A | Genotyped by Kbioscience (http://www.kbioscience.co.uk) | 99% | 99% | 0.16 |
| ERF | rs3751812 | 0.99 | A | Illumina 318K, Illumina 370K, and Affymetrix 250K chips | 95% | NA | 0.03 |
| EYHS | rs1121980 | 0.84 | T | TaqMan® SNP Genotyping Assays (Applied Biosystems) | 97% | 100% | 0.30 |
| FamHS | rs3751812 | 1 | T | Illumina 550K chip | 100% | NA | 0.01 |
| FUSION Stage 1 | rs8050136 | 1 | A | Illumina Infinium Human Hap300 BeadChip | 100% | 100% | 0.16 |
| FUSION Stage 2 | rs9939609 | - | A | Illumina Infinium Human Hap300 BeadChip | 100% | 100% | 0.16 |
| GENDAI | rs9939609 | - | A | iPLEX™ Sequenom MassARRAY® | 98% | 99% | 0.05 |
| GLACIER | rs9939609 | - | A | TaqMan® SNP Genotyping Assays (Applied Biosystems) | >96% | >99 % | 0.11 |
| GOOD | rs3751812 | 1 | T | Illumina HumanHap 610K chip | 100% | 100% | 0.84 |
| HAPI | rs9939609 | - | A | Affymetrix GenChip Human Mapping 500K chip | 99% | NA | 0.58 |
| HELENA | rs9939609 | - | A | Illumina Golden Gate Assay | 100% | NA | 0.56 |
| HUNT2-DiaB | rs9939609 | - | A | iPLEX™ Sequenom MassARRAY® | 100% | 100% | 0.88 |
| InCHIANTI | rs8050136 | 1 | A | Illumina 500K chip | >98% | NA | 0.46 |
| Inter99 | rs9939609 | - | A | TaqMan® SNP Genotyping Assays (Applied Biosystems) | 97% | 100% | 0.73 |
| MDC | rs9939609 | - | A | iPLEX™ Sequenom MassARRAY® or TaqMan® SNP Genotyping Assays (Applied Biosystems) | 97% | 99% | 0.99 |
| METSIM | rs9939609 | - | A | TaqMan® SNP Genotyping Assays (Applied Biosystems) | 100% | 100% | 0.91 |
| MONICA/KORA | rs9935401 | 1 | A | iPLEX™ Sequenom MassARRAY® | 95% | NA | 0.50 |
| MPP | rs9939609 | - | A | TaqMan® SNP Genotyping Assays (Applied Biosystems) | >99 % | >99 % | 0.35 |
| MRC Ely | rs1121980 | 0.84 | T | TaqMan® SNP Genotyping Assays (Applied Biosystems) | >98 % | >99% | 0.85 |

| | | | | | | | |
|---|---|---|---|---|---|---|---|
| NFBC1966 | rs9939609 | - | A | TaqMan® SNP Genotyping Assays (Applied Biosystems) | 88% | 100% | 0.33 |
| NFBC1986 | rs1421085 | 0.93 | C | TaqMan® SNP Genotyping Assays (Applied Biosystems) | 99% | 100% | 1.00 |
| NHS | rs9939609 | - | A | TaqMan® SNP Genotyping Assays (Applied Biosystems) | >95 % | >99% | 0.10 |
| HPFS | rs9939609 | - | A | TaqMan® SNP Genotyping Assays (Applied Biosystems) | >95 % | >99% | 0.03 |
| ORGGEN | rs9939609 | - | A | TaqMan® SNP Genotyping Assays (Applied Biosystems) | 96% | 100% | 0.27 |
| PANIC | rs8050136 | 1 | A | TaqMan® SNP Genotyping Assays (Applied Biosystems) | 100% | 100% | 0.95 |
| PPP-Botnia | rs9939609 | - | A | TaqMan® SNP Genotyping Assays (Applied Biosystems) | >99 % | >99% | 0.37 |
| QFS | rs8050136 | 1 | A | Illumina Golden Gate Assay | NA | >99% | 0.14 |
| RISC | rs9939609 | - | A | Genotyped by Kbioscience (http://www.kbioscience.co.uk) | NA | NA | 0.99 |
| Rotterdam | rs8050136 | 1 | A | Illumina Infinium HumanHap 55 BeadChip v.3. | ≥97 % | 99% | 0.96 |
| Segovia | rs9939609 | - | A | TaqMan® SNP Genotyping Assays (Applied Biosystems) | 95% | 1 | 0.90 |
| Singapore NHS98 | rs9939609 | - | A | iPLEX™ Sequenom MassARRAY® | 98% | 1 | Chinese: 0.65<br>Malay: 0.59<br>Asian Indian: 0.23 |
| STRIP | rs9939609 | - | A | TaqMan® SNP Genotyping Assays (Applied Biosystems) | 97% | 1 | 0.25 |
| TUEF & TULIP | rs8050136 | 1 | A | TaqMan® SNP Genotyping Assays (Applied Biosystems) | NA | NA | 0.95 |
| TwinsUK | rs8050136 | 1 | A | Illumina Infinium Assay | 100% | NA | 0.76 |
| WGHS | rs8050136 | 1 | A | Illumina Infinium II assay (Human HAP300 panel) | >99 % | NA | 0.88 |
| WHI-OS | rs9939609 | - | A | TaqMan® SNP Genotyping Assays (Applied Biosystems) | 98% | 99% | White: 0.17<br>African American:0.16<br>Asian: 0.45<br>Hispanic: 0.15 |
| YFS | rs9939609 | - | A | TaqMan® SNP Genotyping Assays (Applied Biosystems) | 100% | 100% | 0.56 |

NA, data not available; * $r^2$ in the HapMap CEU population
