# Supplementary material for: Physical Activity Attenuates the Influence of FTO Variants on Obesity Risk: A Meta-Analysis of 218,166 Adults and 19,268 Children
Source: PLoS Med. 2011 Nov 1;8(11):e1001116. doi: 10.1371/journal.pmed.1001116 (PMC3206047; doi:10.1371/journal.pmed.1001116)
Supplement: Text S2 — Acknowledgments and funding. (PDF) [file pmed.1001116.s016.pdf]

Physical activity attenuates the influence of *FTO* variants on obesity; a meta-analysis of 218,166 adults and 19,268 children

Tuomas O Kilpeläinen, Lu Qi, Soren Brage, Stephen J Sharp, Emily Sonestedt, *et al.**

* A full list of author names appears in the main paper.

TEXT S2

Acknowledgements **p. 2**

Funding **p. 4**

ACKNOWLEDGEMENTS

ALSPAC (Avon Longitudinal Study of Parents and Children) – We are extremely grateful to all the families who took part in this study, the midwives for their help in recruiting them, and the whole ALSPAC team, which includes interviewers, computer and laboratory technicians, clerical workers, research scientists, volunteers, managers, receptionists and nurses.

ARIC (Atherosclerosis Risk in Communities) –The authors thank the staff and participants of the ARIC study for their important contributions.

Birth Cohort 1958 (1958 British Birth Cohort) –Dr Sue Ring and Dr Wendy McArdle (University of Bristol), and Mr Jon Johnson (Centre for Longitudinal Studies, Institute of Education, London) are thanked for help with data linkage.

BWHHS (British Women's Heart & Health Study) – We would like to thank all of the participants and staff from the British Women's Heart & Health Study.

CLHNS (Cebu Longitudinal Health and Nutrition Survey) – We thank Linda S. Adair, Judith B. Borja, Nanette Lee and the Office of Population Studies Foundation research and data collection teams for the Cebu Longitudinal Health and Nutrition Survey.

CoLaus (Cohorte Lausanneoise) – The authors thank Peter Vollenweider, Gerard Waeber, Meg G. Ehm, Dawn Waterworth, Vincent Mooser, Murielle Bochud, and Jacqui S. Beckmann.

DPS (The Diabetes Prevention Study) – We thank Tiina Lappalainen for genotyping and David Laaksonen for his help with generating the physical activity variable.

ELSA (English Longitudinal Study of Aging) – ELSA was developed by a team of researchers based at University College London, the National Centre for Social Research and the Institute of Fiscal Studies. The data were collected by the National Centre for Social Research.

ERF (Erasmus Rucphen Family Study) – We are grateful to all general practitioners for their contributions, to Petra Veraart for her help in genealogy, Jeannette Vergeer for the supervision of the laboratory work and Peter Snijders for his help in data collection.

GENDAI (Gene-Diet Attica Investigation on Childhood Obesity) – We thank our participating children and their schools for their help and enthusiasm.

GLACIER (Gene-Lifestyle Interactions and Complex Traits Involved in Elevated Disease Risk) – The authors thank the participants in the GLACIER Study, the staff of the Umeå Medical Biobank for preparing the materials, and the Västerbottens Intervention Programme for data collection. The authors also thank K. Enqvist and T. Johansson (Västerbottens County Council, Umeå, Sweden) for DNA preparation and P. Soule, H. Ranu, and D.J. Hunter (Harvard School of Public Health, Boston, MA) for support with genotyping.

HAPI (Heredity and Phenotype Intervention Heart Study) – Additional contributors include Evadnie Rampersaud, PhD; Toni I. Pollin, PhD; Haiqing Shen, PhD; Jeffery R. O'Connell, PhD; Julie L. Ducharme, MD. The authors would like to extend their gratitude to the Amish study participants and to the extraordinary efforts of our Amish Research Clinic and laboratory staff.

HUNT2-DiaB (The HUNT2-Bergen Diabetes Research Collaboration) – We thank Jens K. Hertel, Anders Molven, Kristian Hveem, Carl Platau and Kristian Midthjell in the Norwegian HUNT-Bergen Diabetes Research Collaboration.

Inter99 (Inter99 Population-Based Cohort of Middle-Aged Danes) – The Inter99 project was initiated by: T. Jørgensen (PI), K. Borch-Johnsen (co-PI), H. Ibsen and T. F. Thomsen. The steering committee comprises the former two and C. Pisinger.

MPP (Malmö Preventive Project) – We thank the study participants, the staff at the CRC in Malmö (Anna Berglund, Margareta Svensson, and Malin Neptin et al) for excellent technical assistance, and staff at the Malmö Medical Biobank (Göran Berglund et al).

MRC Ely (The MRC Ely Study) – We thank Sarah Dawson, Farzana Shah, Sofie Ashford, Larissa Richardson, Steven Knighton, and Chris Gillson for their rapid and accurate large-scale sample preparation and genotyping, and Jian'an Luan and Stephen Sharp for their statistical support.

NFBC1966 & NFBC1986 (Northern Finland Birth Cohorts) – The authors thank Professor Paula Rantakallio (launch of the Northern Finland Birth Cohort studies and initial data collection), Outi Tornwall, (DNA biobanking) and Minttu Jussila (DNA biobanking).

ORGGEN (Obesity Research Group – Genetics) – Expert assistance with genotyping provided by Torben Hansen and Oluf Borbye Pedersen is thankfully acknowledged.

PPP-Botnia (Prevalence, Prediction, and Prevention of Diabetes in Botnia Study) – We thank the participants in the study as well as the Botnia Study Group. We are also grateful for the technical assistance of the staff of the CRC in Malmö.

QFS (Quebec Family Study) – Thanks are expressed to Dr Germain Thériault and to Guy Fournier, Lucie Allard, Monique Chagnon and Claude Leblanc for their contributions to the recruitment and data collection of the QFS.

Rotterdam (Rotterdam Study) – We thank Pascal Arp, Mila Jhamai, Dr Michael Moorhouse, Marijn Verkerk, and Sander Bervoets for their help in creating the GWAS database. The authors are very grateful to the participants and staff from the Rotterdam Study, the participating general practioners and the pharmacists.

Segovia (The Segovia Study) – Authors thank the members of the Segovia Insulin Resistance Study Group. Authors also thank Dr Cristina Fernández-Pérez for the statistical advice.

STRIP (The Special Turku Coronary Risk Factor Intervention Project for Children) – We wish to thank prof. Olli Simell and other members of the STRIP Study Group, especially Maiju Saarinen, Ms.Sci, for performing the statistical analyses of the STRIP Study.

TwinsUK – Genotyping of TwinsUK samples: We thank the staff from the Genotyping Facilities at the Wellcome Trust Sanger Institute for sample preparation, Quality Control and Genotyping led by Leena Peltonen and Panos Deloukas; Le Centre National de Génotypage, France, led by Mark Lathrop, for genotyping; Duke University, North Carolina, USA, led by David Goldstein, for genotyping; and the Finnish Institute of Molecular Medicine, Finnish Genome Center, University of Helsinki, led by Aarno Palotie.

WGHS (Women's Genome Health Study) – The authors thank the investigators, staff, and participants of the Women's Genome Health Study for their valuable contributions. The authors would like to thank Lynda Rose and Latha Padmanabhan for their help with the statistics.

WHI-OS (The Women's Health Initiative – Observational Study) – The authors thank the WHI investigators and staff for their dedication, and the study participants for making the program possible.

YFS (The Cardiovascular Risk in Young Finns Study) – The expert technical assistance in the statistical analyses by Irina Lisinen and Ville Aalto is gratefully acknowledged.

FUNDING

AGES-Reykjavik (Age, Gene/Environment Susceptibility Reykjavik Study) – The Age, Gene/Environment Susceptibility-Reykjavik Study is funded by National Institutes of Health contract N01-AG-12100, the National Institute of Aging Intramural Research Program, Hjartavernd (the Icelandic Heart Association), and the Althingi (the Icelandic Parliament).

ALSPAC (Avon Longitudinal Study of Parents and Children) – The UK Medical Research Council, the Wellcome Trust and the University of Bristol provide core support for ALSPAC.

ARIC (Atherosclerosis Risk in Communities) – The Atherosclerosis Risk in Communities (ARIC) Study is carried out as a collaborative study supported by National Heart, Lung, and Blood Institute contracts N01-HC-55015, N01-HC-55016, N01-HC-55018, N01-HC-55019, N01-HC-55020, N01-HC-55021, N01-HC-55022, R01HL087641, R01HL59367 and R01HL086694; National Human Genome Research Institute contract U01HG004402; and National Institutes of Health contract HHSN268200625226C. Infrastructure was partly supported by Grant Number UL1RR025005, a component of the National Institutes of Health and NIH Roadmap for Medical Research.

Birth Cohort 1958 (1958 British Birth Cohort) – Dr Elina Hyppönen holds the Department of Health (UK) Public Health Career Scientist Award. Analyses were funded by the British Heart Foundation (PG/09/023) and as part of the Public Health Research Consortium (supported by the Department of Health Policy Research Programme). The views expressed in the publication are those of the authors and not necessarily those of the Department of Health. Information about the wider programme of the PHRC is available from www.york.ac.uk/phrc. Collection of DNA in the 1958 Birth Cohort was funded by the Medical Research Council grant G0000934 and Wellcome Trust grant 068545/Z/02. Genotyping was carried out in the Juvenile Diabetes Research Foundation/Wellcome Trust Diabetes and Inflammation Laboratory, Cambridge Institute for Medical Research, University of Cambridge, UK (Led by Professor John Todd). This work was undertaken at Great Ormond Street Hospital /University College London, Institute of Child Health which received a proportion of funding from the Department of Health's National Institute of Health Research ('Biomedical Research Centres' funding). The Medical Research Council provides funds for the MRC Centre of Epidemiology for Child Health.

BLSA (Baltimore Longitudinal Study of Aging) – This study was supported in part by the Intramural Research Program of the NIH, National Institute on Aging. A portion of that support was through a R&D contract with MedStar Research Institute.

BWHHS (British Women's Heart & Health Study) – The British Women's Heart & Health Study is jointly funded by the UK Department of Health Policy Research Programme and the British Heart Foundation. The MRC provides funds for the MRC Centre for Causal Analyses in Translational Epidemiology.

CLHNS (Cebu Longitudinal Health and Nutrition Survey) – This work was supported by National Institutes of Health grants DK078150 (Karen L. Mohlke), TW05596 (Linda S. Adair), HL085144 (Linda S. Adair), TW008288 (Nanette Lee) and pilot funds from RR20649, ES10126, and DK56350.

CoLaus (Cohorte Lausanneoise) – The CoLaus study received financial contributions from GlaxoSmithKline, the Faculty of Biology and Medicine of Lausanne, and the Swiss National Science Foundation (33CSCO-122661).

DESIR (Data from an Epidemiological Study on the Insulin Resistance Syndrome) – This work was supported by the French Government ("Agence Nationale de la Recherche"), the French Region of "Nord Pas De Calais" ("Contrat de Projets État-Région"), and the charities: "Association Française des Diabétiques", "Programme National de Recherche sur le Diabète" and "Association de Langue Française pour l'Etude du Diabète et des Maladies Métaboliques". The D.E.S.I.R. study has been supported by CNAMTS, Lilly, Novartis Pharma and Sanofi-Aventis, by INSERM ("Réseaux en Santé Publique, Interactions entre les déterminants de la santé"), Cohortes "Association Diabète Risque Vasculaire",

"Fédération Française de Cardiologie", "Fondation de France", ALFEDIAM, ONIVINS, Ardix Medical, Bayer Diagnostics, Becton Dickinson, Cardionics, Merck Santé, Novo Nordisk, Pierre Fabre, Roche, Topcon. The D.E.S.I.R. Study Group: INSERM U1018: B. Balkau, P. Ducimetière, E. Eschwège; INSERM U367: F. Alhenc-Gelas; CHU D'Angers: Y. Gallois, A. Girault; Bichat Hospital: F. Fumeron, M. Marre, R. Roussel; CHU Rennes: F. Bonnet; CNRS UMR8090, Lille: P. Froguel; Medical Examination Services: Alençon, Angers, Blois, Caen, Chartres, Chateauroux, Cholet, Le Mans, Orleans and Tours; Research Institute for General Medicine: J. Cogneau; General practitioners of the region; Cross-Regional Institute for Health: C. Born, E. Caces, M. Cailleau, O. Lantieri, J. G. Moreau, F. Rakotozafy, J. Tichet, S. Vol. This study was supported in part by a grant from the European Union (Integrated Project EuroDia LSHM-CT-2006-518153 in the Framework Programme 6 [FP6] of the European Community).

DPP (The Diabetes Prevention Program) – The NIDDK of the National Institutes of Health provided funding to the clinical centers and the Coordinating Center for the design and conduct of the study; collection, management, analysis, and interpretation of the data. The Southwestern American Indian Centers were supported directly by the NIDDK and the Indian Health Service. The General Clinical Research Center Program, National Center for Research Resources supported data collection at many of the clinical centers. Funding for data collection and participant support was also provided by the Office of Research on Minority Health, the National Institute of Child Health and Human Development, the National Institute on Aging, the Centers for Disease Control and Prevention, Office of Research on Women's Health, and the American Diabetes Association. Bristol-Myers Squibb and Parke-Davis provided medication. This research was also supported, in part, by the intramural research program of the NIDDK. LifeScan Inc., Health O Meter, Hoechst Marion Roussel, Inc., Merck-Medco Managed Care, Inc., Merck and Co., Nike Sports Marketing, Slim Fast Foods Co., and Quaker Oats Co. donated materials, equipment, or medicines for concomitant conditions. McKesson BioServices Corp., Matthews Media Group, Inc., and the Henry M. Jackson Foundation provided support services under subcontract with the Coordinating Center. The opinions expressed are those of the investigators and do not necessarily reflect the views of the Indian Health Service or other funding agencies. This work was also funded by R01 DK072041 to David Altshuler, Jose C. Florez and Kathleen A. Jablonski. JCF is also supported by the Massachusetts General Hospital and a Clinical Scientist Development Award by the Doris Duke Charitable Foundation.

DPS (The Diabetes Prevention Study) – This work was funded by the Academy of Finland and Finnish Diabetes Research Foundation.

ELSA (English Longitudinal Study of Aging) – Samples from the English Longitudinal Study of Ageing (ELSA) DNA Repository (EDNAR), received support under a grant (AG1764406S1) awarded by the National Institute on Aging (NIA).

EPIC-NL (The European Prospective Investigation into Cancer and Nutrition – Netherlands Study) – The EPIC-NL study was funded by 'Europe against Cancer' Programme of the European Commission (SANCO), Dutch Ministry of Public Health, Welfare and Sports (VWS), Netherlands Cancer Registry (NKR), LK Research Funds, Dutch Prevention Funds, Dutch Cancer Society; ZonMW the Netherlands Organisation for Health Research and Development, and World Cancer Research Fund (WCRF) (The Netherlands). Genotyping of the IBC-chip was funded by IOP Genomics grant IGE05012 from NL Agency.

EPIC-Norfolk (The European Prospective Investigation into Cancer and Nutrition – Norfolk Study) – The EPIC Norfolk Study is funded by program grants from the Medical Research Council UK and Cancer Research UK; and by additional support from the European Union; Stroke Association; British Heart Foundation; Department of Health; Food Standards Agency; and the Wellcome Trust. IB acknowledges support from EU FP6 funding (contract no LSHM-CT-2003-503041).

EPIC-Potsdam (The European Prospective Investigation into Cancer and Nutrition – Potsdam Study) – The recruitment phase of the EPIC-Potsdam Study was supported by the German Federal Ministry of Research and Technology (01 EA 9401) and the European Union (SOC 95201408 05F02). This work was funded by the German National Genome Research Net NGFN2 (grant 01GS0487) and the German Federal Ministry of Education and Research (BMBF32007).

ERF (Erasmus Rucphen Family Study) – The study was supported by grants from The Netherlands Organisation for Scientific Research (NWO), Erasmus MC and the Centre for Medical Systems Biology (CMSB).

EYHS (European Youth Heart Study) – The EYHS was supported by grants from the following agencies: The Danish Heart Foundation, The Danish Medical Research Council Health Foundation, The Danish Council for Sports Research, The Foundation in Memory of Asta Florida Bolding Renée Andersen, The Faculty of Health Sciences, University of Southern Denmark, and The Estonian Science Foundation (grant numbers 3277 and 5209).

FamHS (Family Heart Study) – The FamHS is funded by a NHLBI grant 5R01HL08770003, and NIDDK grants 5R01DK06833603 and 5R01DK07568102.

FUSION (Finland-United States Investigation of NIDDM Genetics Study) – Support for this research was provided by NIH grants DK062370 (MB), DK072193 (KLM), and National Human Genome Research Institute intramural project number 1 Z01 HG000024 (FSC).

GENDAI (Gene-Diet Attica Investigation on Childhood Obesity) – The collection of the GENDAI cohort was funded by Coca-Cola Hellas, whereas the genotyping was funded by NIDDK K23 DK067288 awarded to Joel N. Hirschhorn.

GLACIER (Gene-Lifestyle Interactions and Complex Traits Involved in Elevated Disease Risk) – The GLACIER Study was funded by project grants from Novo Nordisk, the Swedish Heart-Lung Foundation, the Swedish Diabetes Association, the Påhlssons Foundation, the Swedish Research Council, the Umeå Medical Research Foundation, and The Heart Foundation of Northern Sweden (all to P.W.F.). F.R. was supported by a postdoctoral stipend from the Swedish Heart-Lung Foundation.

GOOD (Gothenburg Osteoporosis and Obesity Determinants Study) – Financial support for the GOOD study was received from the Swedish Research Council, the Swedish Foundation for Strategic Research, The ALF/LUA research grant in Gothenburg, the Lundberg Foundation, the Emil and Vera Cornell Foundation, the Torsten and Ragnar Söderberg's Foundation, Petrus and Augusta Hedlunds Foundation, the Västra Götaland Foundation, the Göteborg Medical Society, and the Sahlgrenska Center for Cardiovascular and Metabolic Research (CMR, no. A305:188), which is supported by the Swedish Strategic Foundation.

HAPI (Heredity and Phenotype Intervention Heart Study) – The HAPI study receives funding from NIH grant U01 HL072515. Partial funding for this study was provided by the Clinical Nutrition Research Unit of Maryland, grant P30 DK072488; the University of Maryland General Clinical Research Center, grant M01 RR 16500; the Johns Hopkins University General Clinical Research Center, grant M01 RR 000052; the Geriatric Research and Education Clinical Center, Baltimore Veterans Administration Medical Center; and the American Diabetes Association. Dr. Rampersaud was funded by a postdoctoral NIH/NHLBI sponsored NRSA training grant T32HL072751.

HELENA (Healthy Lifestyle in Europe by Nutrition in Adolescence) – The HELENA study takes place with the financial support of the European Community Sixth RTD Framework Programme (Contract FOOD-CT-2005-007034). This study is also being supported by the Swedish Council for Working Life and Social Research (FAS) and Spanish Ministry of Science and Innovation (RYC-2010-05957).

HUNT2-DiaB (The HUNT2-Bergen Diabetes Research Collaboration) – We thank for support from the University of Bergen, the Research Council of Norway, Helse Vest, Innovest, the HUNT Research Center, the Norwegian University of Science and Technology, the Norwegian Institute for Public Health and the Nord-Trøndelag County Council.

InCHIANTI (InCHIANTI Study) – The InCHIANTI study baseline (1998-2000) was supported as a "targeted project" (ICS110.1/RF97.71) by the Italian Ministry of Health and in part by the U.S. National Institute on Aging (Contracts: 263 MD 9164 and 263 MD 821336).

Inter99 (Inter99 Population-Based Cohort of Middle-Aged Danes) – The Inter99 project was financially supported by research grants from the Danish Research Council, The Danish Centre for Health Technology Assessment, Novo Nordisk Inc., Research Foundation of Copenhagen County, Ministry of Internal Affaires and Health, The Danish Heart Foundation,The Danish Pharmaceutical Association, The Augustinus Foundation, The Ib Henriksen Foundation, and the Becket Foundation.

KORA Studies (Cooperative Health Research in the Region of Augsburg, KOoperative Gesundheitsforschung in der Region Augsburg) – The KORA research platform and the MONICA Augsburg studies were initiated and financed by the Helmholtz Center München, German Research Center for Environmental Health, which is funded by the German Federal Ministry of Education and Research and by the State of Bavaria. The work of KORA is supported by the German Federal Ministry of Education and Research (BMBF) in the context of the German National Genome Research Network (NGFN-2 and NGFN-plus). Our research was supported within the Munich Center of Health Sciences (MC Health) as part of LMUinnovativ.

MDC (Malmö Diet and Cancer Cohort) – MDC is supported by the Swedish Medical Research Council, equipment grant from the Knut and Alice Wallenberg Foundation, the Swedish Heart and Lung Foundation, The Swedish Diabetes Foundation, the Region Skåne, the Malmö University Hospital, the Albert Påhlsson Research Foundation, the Crafoord foundation, and the Lund University Diabetes Center (LUDC).

METSIM (The Metabolic Syndrome in Men Study) – The METSIM Study is funded by grants from the Academy of Finland (Contract no. 124243), The Finnish Heart Foundation, The Finnish Diabetes Foundation, TEKES (Contract no. 1510/31/06), and Commission of the European Community (LSHM-CT-2004-512013 EUGENE2, and HEALTH-F2-2007-201681).

MPP (Malmö Preventive Project) – This work was funded in part by grants from the Swedish Diabetes Association, Swedish Heart Lung-Foundation, Novo Nordisk, the Knut and Alice Wallenberg Foundation, the Påhlsson Foundation, the Sigrid Juselius Foundation, the Folkhälsan Research Foundation, the Nordic Center of Excellence in Disease Genetics, an EU grant (EXGENESIS), and the Signe and Ane Gyllenberg Foundation.

MRC Ely (The MRC Ely Study) – The MRC Ely Study was funded by the Medical Research Council and the Wellcome Trust.

NHS & HPFS (Nurses Health Study & Health Professionals Follow-Up Study) – Lu Qi is supported by National Institutes of Health grants RO1 HL71981, American Heart Association Scientist Development Award and the Boston Obesity Nutrition Research Center (DK46200).

NFBC1966 & NFBC1986 (Northern Finland Birth Cohorts) – Financial support was received from the Academy of Finland (project grants 104781 and 120315 and the Center of Excellence in Complex Disease Genetics); University Hospital Oulu (Oulu, Finland); Biocenter Oulu, University of Oulu (Oulu, Finland); the European Commission (European Birth-Life Course Studies (EURO-BLCS), Framework 5 award QLG1-CT-2000-01643); the US National Heart, Lung, and Blood Institute (grant 5R01HL087679-02), through the STAMPEED Program (grant 1RL1MH083268-01); the US National Institute of Mental Health (grant 5R01MH63706:02); the ENGAGE Project (grant HEALTH-F4-2007-201413); and the United Kingdom Medical Research Council (studentship grant G0500539). DNA extraction, sample quality control, biobank upkeep, and aliquoting were performed at the National Public Health Institute, Biomedicum Helsinki (Helsinki, Finland); these activities were supported financially by the Academy of Finland and Biocentrum Helsinki.

PANIC (The Physical Activity and Nutrition in Children Study) – The PANIC Study has been financially supported by Ministry of Social Affairs and Health of Finland (178/THL/TE/2010), Ministry of Education and Culture of Finland (121/627/2009), Finnish Innovation Fund Sitra, Social Insurance Institution of Finland (22/26/2008), Finnish Cultural Foundation, Juho Vainio

Foundation, Foundation for Paediatric Research, University of Eastern Finland and Kuopio University Hospital (5031343).

PPP-Botnia (Prevalence, Prediction, and Prevention of Diabetes in Botnia Study) – This work was funded in part by grants from the Swedish Diabetes Association, Swedish Heart Lung-Foundation, Novo Nordisk, the Knut and Alice Wallenberg Foundation, the Påhlsson Foundation, the Sigrid Juselius Foundation, the Folkhälsan Research Foundation, the Nordic Center of Excellence in Disease Genetics, an EU grant (EXGENESIS), the Signe and Ane Gyllenberg Foundation, the Swedish Cultural Foundation in Finland, the Finnish Diabetes Research Foundation, the Foundation for Life and Health in Finland, the Finnish Medical Society, the Paavo Nurmi Foundation, Helsinki University Central Hospital Research Foundation, the Perklén Foundation, the Ollqvist Foundation and the Närpes Health Care Foundation.

QFS (Quebec Family Study) – The Quebec Family Study was supported over the years by multiple grants from the Medical Research Council of Canada and the Canadian Institutes for Health Research (PG-11811, MT-13960 and GR-15187) as well as other agencies.

RISC (Relationship between Insulin Sensitivity and Cardiovascular Disease) – The RISC study was funded by European Union Grant QLG1-CT-2001-01252 and AstraZeneca.

Rotterdam (Rotterdam Study) – The GWA study was funded by the Netherlands Organisation of Scientific Research NWO Investments (nr. 175.010.2005.011, 911-03-012), the Research Institute for Diseases in the Elderly (014-93-015; RIDE2), the Netherlands Genomics Initiative (NGI)/Netherlands Organisation for Scientific Research (NWO) project nr. 050-060-810. The Rotterdam Study is funded by Erasmus Medical Center and Erasmus University, Rotterdam, Netherlands Organization for the Health Research and Development (ZonMw), the Research Institute for Diseases in the Elderly (RIDE), the Ministry of Education, Culture and Science, the Ministry for Health, Welfare and Sports, the European Commission (DG XII), and the Municipality of Rotterdam. We would like to thank Dr. Tobias A. Knoch, Luc V. de Zeeuw, Anis Abuseiris, and Rob de Graaf as well as their institutions the Erasmus Computing Grid, Rotterdam, The Netherlands, and especially the national German MediGRID and Services@MediGRID part of the German D-Grid, both funded by the German Bundesministerium für Forschung und Technology under grants #01 AK 803 A-H and # 01 IG 07015 G for access to their grid resources.

Segovia (The Segovia Study) – This work was supported by CIBER de Diabetes y Enfermedades Metabólicas Asociadas, an ISCIII project, Madrid. Spain. A partial support also came from Educational Grants from Eli Lilly Laboratory, Spain.

Singapore NHS98 (1998 Singapore National Health Survey) – Financial support for this work was provided by the Singapore Biomedical Research Council (05/1/36/19/413).

TUEF & TULIP (Tuebingen Family Study & Tuebingen Lifestyle Intervention Programme) – The studies were supported by a grant from the Deutsche Forschungsgemeinschaft (KFO 114).

TwinsUK – The study was funded by the Wellcome Trust; European Community's Seventh Framework Programme (FP7/2007-2013)/grant agreement HEALTH-F2-2008-201865-GEFOS and (FP7/2007-2013), ENGAGE project grant agreement HEALTH-F4-2007-201413 and the FP-5 GenomEUtwin Project (QLG2-CT-2002-01254). The study also receives support from the Dept of Health via the National Institute for Health Research (NIHR) comprehensive Biomedical Research Centre award to Guy's & St Thomas' NHS Foundation Trust in partnership with King's College London. TDS is an NIHR senior Investigator. The project also received support from a Biotechnology and Biological Sciences Research Council (BBSRC) project grant. (G20234). The authors acknowledge the funding and support of the National Eye Institute via an NIH/CIDR genotyping project (PI: Terri Young).

WGHS (Women's Genome Health Study) – T.A. was supported by NHLBI (T32HL07575). S.M. was supported by NHLBI (K08 HL094375). WGHS is supported by grants HL-43851 and CA-47988 from the

NHLBI and the NCI. Genotyping was provided by Amgen, Inc (Cambridge, MA). The funding agencies played no role in the design, conduct, data management, analysis, or manuscript preparation related to this manuscript.

WHI-OS (The Women's Health Initiative – Observational Study) – The WHI program is funded by the National Heart, Lung, and Blood Institute, National Institutes of Health, U.S. Department of Health and Human Services through contracts N01WH22110, 24152, 32100-2, 32105-6, 32108-9, 32111-13, 32115, 32118-32119, 32122, 42107-26, 42129-32, and 44221.

YFS (The Cardiovascular Risk in Young Finns Study) – The Young Finns Study has been financially supported by the Academy of Finland: grants 126925, 121584, 124282, 129378 (Salve), 117787 (Gendi), and 41071 (Skidi), the Social Insurance Institution of Finland, Kuopio, Tampere (grants for TL and MK) and Turku University Hospital Medical Funds, Juho Vainio Foundation, Paavo Nurmi Foundation, Finnish Foundation of Cardiovascular Research and Finnish Cultural Foundation.
